# Improving respiratory and heart rate variation estimation from resting-state BOLD fMRI across the lifespan using a functionally informed, tissue-aware deep learning framework

**DOI:** 10.1162/IMAG.a.1346

**Published:** 2026-08-27

**Authors:** Abdoljalil Addeh, Karen Ardila, Pattarawut Charatpangoon, Ethan Church, Rebecca J. Williams, G. Bruce Pike, M. Ethan MacDonald

**Affiliations:** Department of Biomedical Engineering, Schulich School of Engineering, University of Calgary, Calgary, Canada; Department of Electrical & Software Engineering, Schulich School of Engineering, University of Calgary, Calgary, Canada; Department of Radiology, Cumming School of Medicine, University of Calgary, Calgary, Canada; Hotchkiss Brain Institute, Cumming School of Medicine, University of Calgary, Calgary, Canada; Department of Computer Science, McGill University, Montréal, Canada; Brain-Behaviour Research Group, University of New England, Armidale, Australia; Department of Clinical Neurosciences, Cumming School of Medicine, University of Calgary, Calgary, Canada

**Keywords:** fMRI, head motion parameters, heart rate variation, respiratory variation, dynamic functional connectivity

## Abstract

Accurate measurement of physiological signals such as respiration and cardiac activity is essential for modeling physiological confounds in BOLD-fMRI data. However, external monitoring devices such as respiratory belts and photoplethysmographs often suffer from signal loss, noise, or incomplete recordings, and many fMRI datasets lack physiological measurements altogether due to practical constraints. In this work, we propose a data-driven framework to reconstruct respiratory variation (RV) and heart rate variation (HRV) directly from resting-state fMRI data using a hybrid machine learning architecture that combines one-dimensional convolutional neural networks (1D-CNN) with gated recurrent units (GRUs). The model processes BOLD signals from 630 regions of interest (ROIs), spanning cortical and subcortical gray matter, white matter, and cerebrospinal fluid. For RV estimation, six head motion parameters were included as additional inputs to capture motion-related physiological information. The proposed method was trained and evaluated on three cohorts across the lifespan from the Human Connectome Project (HCP), including HCP in Development (HCP-D, ages 5–21 years), HCP in Young Adults (HCP-YA, ages 22–35 years), and HCP in Aging (HCP-A, ages 36–100 years). Age-specific architectural tuning was applied to accommodate developmental, neurovascular, and physiological variability across these populations. When compared against established CNN- and RNN-based baselines under matched preprocessing and native temporal resolution, the proposed architecture achieved consistent performance gains across all evaluation metrics. The largest relative improvements were observed for error-based and temporal alignment metrics, with MAE, MSE, and DTW reduced by approximately 7–10%, indicating lower absolute reconstruction error and improved temporal fidelity, while correlation-based improvements were more modest (approximately 5–7%). These results demonstrate the feasibility and effectiveness of reconstructing RV and HRV directly from fMRI time series using spatially and temporally enriched neural architectures. Building on earlier efforts in this domain, the proposed framework extends prior work through evaluation across multiple cohorts, broader anatomical coverage, and enhanced modeling capacity, offering a robust alternative for physiological modeling in the absence of external recordings.

## 1 Introduction

Functional magnetic resonance imaging (fMRI) is a widely used neuroimaging modality for investigating brain function and connectivity, both in task-based and resting-state paradigms. The blood oxygen level-dependent (BOLD) signal, which underlies most fMRI studies, reflects changes in cerebral hemodynamics and oxygenation, yet it is also susceptible to a variety of non-neuronal physiological influences (Addeh, Williams, et al., 2025). Among these, low-frequency fluctuations in respiration and heart rate act as major confounds that can significantly impact the amplitude and temporal dynamics of the BOLD signal (Birn et al., 2008; Chang et al., 2009; Chang & Glover, 2009; Golestani et al., 2015; Golestani & Chen, 2020; Wise et al., 2004). These fluctuations introduce spatially and temporally structured noise that can bias activation maps, distort functional connectivity estimates, and reduce statistical sensitivity (Murphy et al., 2013).

Traditional correction methods rely on external physiological monitoring devices, such as respiratory belts and photoplethysmographs (PPG), to record respiratory and cardiac waveforms. From these waveforms, indices such as respiratory variation (RV) and heart rate variation (HRV) are extracted and used in model-based correction techniques to mitigate physiological confounds in BOLD-fMRI data (Chang et al., 2009). However, the utility of these approaches is often constrained by technical and practical limitations. In large-scale neuroimaging studies, physiological recordings are frequently missing, corrupted, or of poor quality due to equipment malfunction, suboptimal sensor placement, participant movement, or time restrictions during scanning. For example, Addeh et al. (2023) reported that up to 87% of the respiratory traces in the Human Connectome Project in Development (HCP-D) dataset were unusable, a limitation that similarly affects other publicly available datasets. These challenges underscore the need for robust, data-driven alternatives independent of external physiological recordings.

In recent years, data-driven machine learning models have emerged as promising alternatives for reconstructing physiological waveforms directly from BOLD-fMRI data, thereby circumventing the dependency on external sensors. Recent work has established the potential of machine learning models for reconstructing RV and HRV directly from BOLD-fMRI (Addeh et al., 2023; Addeh, Vega, et al., 2025; Bayrak et al., 2020, 2021; Salas et al., 2021), and more recently, transformer-based architectures have been introduced to model longer-range temporal dependencies using attention mechanisms (Wang et al., 2025). However, many existing approaches remain limited by narrow frequency bands, reduced temporal resolution due to downsampling, or restricted spatial inputs derived primarily from gray matter, potentially attenuating higher-frequency physiological fluctuations that may contain biologically relevant information. Additionally, a growing line of research has begun to consider head motion parameters, traditionally treated as nuisance regressors, as potential sources of physiological information. Because head motion can capture both true displacement and physiological pseudomotion related to respiration and cardiac pulsatility, it has been proposed as a valuable and still underutilized source of non-neuronal information in fMRI analysis (Addeh, Vega, et al., 2025).

Many physiological processes exhibit both short-term fluctuations and long-range temporal dependencies, requiring models that can capture dynamics across multiple time scales (Berntson et al., 1997; Ivanov et al., 1999; Shmueli et al., 2007; Van den Aardweg & Karemaker, 2002). For instance, Van den Aardweg & Karemaker (2002) observed low-frequency oscillations in end-tidal CO_2_ and mean inspiratory flow with cycle durations ranging from approximately 20 to 60 s, driven by delayed chemoreflex feedback loops. Similarly, Shmueli et al. (2007) demonstrated that fluctuations in heart rate correlate with BOLD signal changes at both short delays (6–12 s) and longer lags of around 30–42 s, highlighting the presence of temporally extended physiological influences on brain activity. One-dimensional convolutional neural networks (1D-CNNs) are effective at identifying short-duration patterns within time series data, while gated recurrent units (GRUs), a streamlined form of recurrent neural networks (RNN), are well suited for modeling sequential dependencies over longer time windows. Compared with traditional recurrent neural networks, GRUs are more computationally efficient and help mitigate the vanishing gradient problem, making them suitable for learning temporal dynamics in physiological signals such as those embedded in BOLD-fMRI signals. However, applying such models uniformly across different age groups remains challenging, as physiological and neurovascular characteristics, such as heart rate, respiratory variability, vascular compliance, and motion artifacts, vary considerably with age.

Building on these insights, we developed a hybrid deep learning architecture that integrates 1D-CNNs with GRUs to reconstruct RV and HRV waveforms directly from resting-state BOLD-fMRI data. The proposed model processes time series from 630 regions of interest (ROIs), encompassing cortical, subcortical, white matter (WM), and cerebrospinal fluid (CSF) regions. For RV reconstruction, the model incorporates six head motion parameters, consistent with our prior work demonstrating their relevance for capturing respiration-related pseudomotion. Our method is evaluated across the full lifespan using three major neuroimaging cohorts, including HCP-D (children) (Harms et al., 2018), HCP in young adults (HCP-YA) (Van Essen et al., 2013), and HCP in Aging (HCP-A) (Harms et al., 2018), with age-specific architectural adaptations to account for differences in neurovascular dynamics, motion characteristics, and physiological signal properties. By combining spatially diverse inputs with temporally enriched modeling, our framework addresses key limitations of prior methods and provides a robust alternative for physiological signal reconstruction in the absence of external recordings.

We hypothesize that respiratory and cardiac fluctuations can be accurately reconstructed from BOLD-fMRI data using a temporally aware, spatially informed neural architecture. Furthermore, we expect that incorporating tissue-specific ROIs and head motion parameters will enhance estimation accuracy across developmental and aging populations.

Specifically, we anticipate that our hybrid 1D-CNN + GRU framework will outperform conventional CNN- and RNN-based models, evaluated under matched preprocessing, temporal resolution, and computationally practical input settings, by more than 5% across evaluation metrics, including mean absolute error (MAE), mean squared error (MSE), Pearson correlation coefficient (r), and dynamic time warping (DTW).

## 2 Method

### 2.1 Dataset

In this study, resting-state fMRI data from three large-scale lifespan cohorts (HCP-D, HCP-YA, and HCP-A) were used for training and testing the proposed model. These datasets collectively span a wide age range (5 to 100 years), enabling the evaluation of model performance across developmental, adult, and aging populations.

Subsequent validation was performed on two additional independent datasets, referred to herein as Dataset 1 (Zvolanek et al., 2023) and Dataset 2 (Spreng et al., 2022), to examine model performance under different acquisition protocols and experimental conditions beyond the HCP cohorts. As the study involved only secondary analysis of anonymized data, no additional institutional ethics approval was required.

Detailed information regarding datasets, subject inclusion, physiological signal quality control, and the fMRI preprocessing pipeline, which includes motion correction, spatial normalization, and ROI time series extraction, is provided in Supplementary Materials, Section 1.

### 2.2 Method

In this study, we introduce a machine learning-based framework to reconstruct RV and HRV waveforms directly from resting-state BOLD-fMRI data. Our approach integrates 1D-CNNs with GRUs, leveraging spatially localized and temporally structured information from BOLD signals together with motion-based physiological cues.

This framework is grounded in several physiological and methodological considerations. Respiratory depth and rate fluctuations are known to influence the BOLD signal with a temporal delay, such that future BOLD samples provide meaningful information for reconstructing current RV. These effects peak approximately 16 s following a deep breath, reflecting the time required for respiration-induced changes in arterial CO_2_ to modulate cerebral blood flow and, consequently, the BOLD signal (Birn et al., 2006). Comparable hemodynamic delays have been observed in breath-hold paradigms, with reported latencies ranging from 10 to 20 s depending on vascular properties and measurement location (Kastrup et al., 1998). Similarly, low-frequency heart rate fluctuations modulate the BOLD signal with characteristic delays, with primary effects peaking at approximately 6–12 s and secondary components emerging around 30–42 s, motivating the use of temporally offset BOLD information for HRV estimation (Shmueli et al., 2007). In addition, both respiratory and cardiac processes exhibit temporal continuity and short- and long-term dependencies, such that past physiological states influence current RV and HRV, and, therefore, past BOLD samples linked to earlier events contribute to reconstruction accuracy (Berntson et al., 1997; Modarreszadeh & Bruce, 1994; Van den Aardweg & Karemaker, 2002).

Beyond cortical signals, non-gray matter tissues provide physiologically informative BOLD fluctuations. Signals from cerebrospinal fluid regions predominantly reflect physiological processes with minimal neural contribution, while white matter BOLD signals are largely, though not exclusively, dominated by non-neuronal sources in resting-state fMRI (Behzadi et al., 2007), making both tissue classes valuable for modeling physiological confounds. Involuntary respiration-related head movements, such as nodding or deep breathing, are captured in estimated head-motion parameters across all six degrees of freedom and frequently oscillate at the respiratory frequency, providing an additional indicator of underlying respiratory activity (Addeh, Vega, et al., 2025; Power et al., 2019). Moreover, respiratory cycles introduce pseudomotion artifacts into motion estimates, particularly in multiband acquisitions, that are present across all motion parameters and can be exploited to infer both respiration depth and rate (Fair et al., 2020; Kaplan et al., 2022). Finally, functional regions defined using dynamic functional connectivity capture transient brain interactions that static atlases cannot, enabling identification of finer and more physiologically meaningful subregions. As a result, dFC-based atlases such as the Dynamic Brain Functional Atlas (D-BFA) provide enhanced sensitivity to physiological fluctuations in BOLD signals (Peng et al., 2023).

To reconstruct RV and HRV, the model processes BOLD time series extracted from a total of 630 ROIs. These include 518 cortical ROIs and 62 subcortical ROIs derived from the Dynamic Brain Functional Atlas (D-BFA; Peng et al. (2023)), 48 white matter ROIs from the Johns Hopkins University DTI-based atlas (Hua et al., 2008), and 2 CSF ROIs corresponding to the lateral ventricles from the Harvard–Oxford subcortical atlas (Makris et al., 2006). This multi-tissue ROI selection enables the model to capture physiologically informative BOLD fluctuations across cortical, subcortical, white matter, and CSF compartments.

Mean BOLD signals within each ROI are used as inputs to improve signal-to-noise ratio (SNR) and reduce computational complexity. The estimation of RV also incorporates six head motion parameters (translations and rotations along the x, y, z axes, pitch, yaw, and roll), consistent with prior work (Addeh, Vega, et al., 2025), whereas HRV estimation relies solely on BOLD signals.

#### 2.2.1 RV reconstruction

**Fig. 1.**
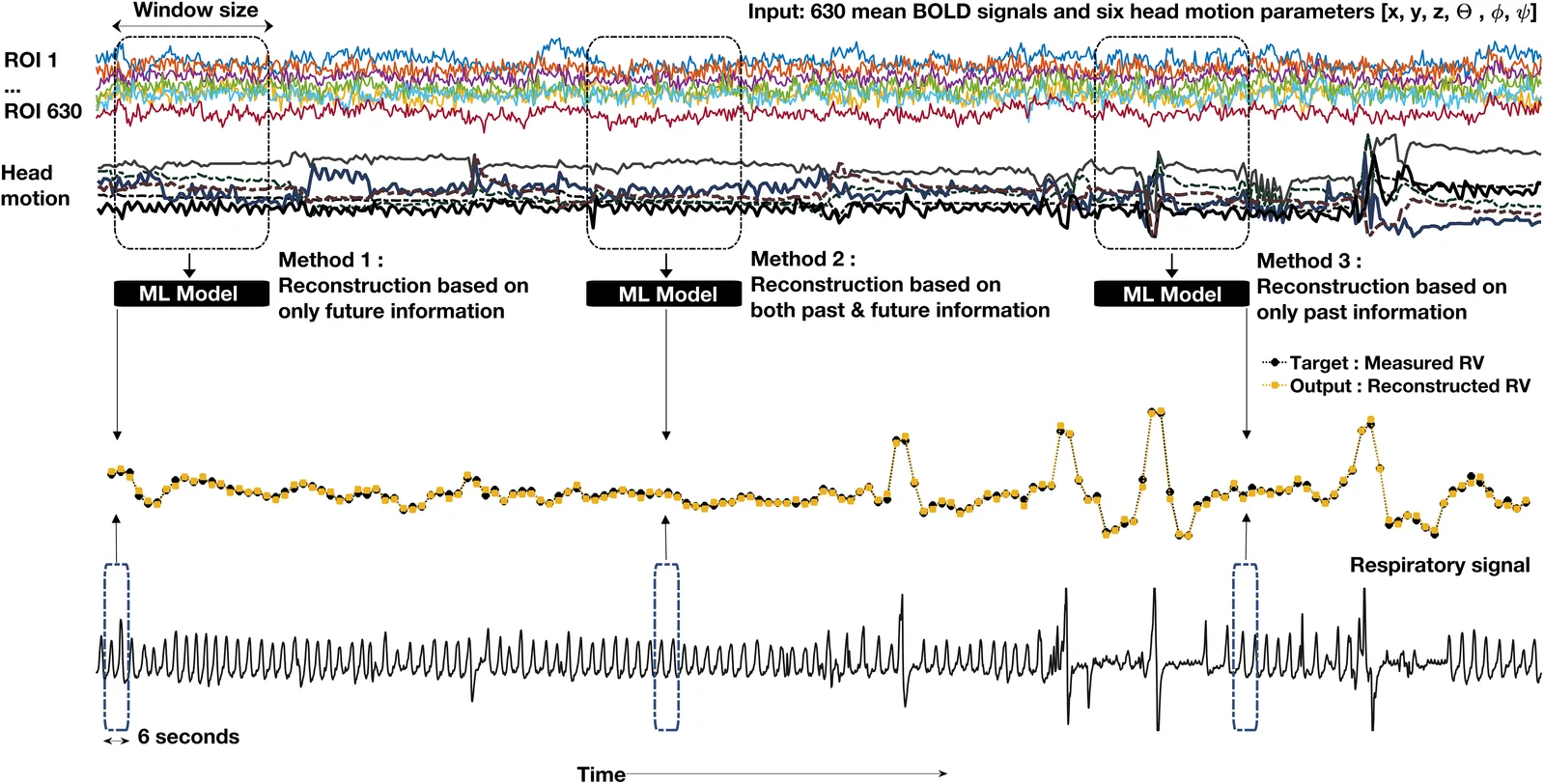
Illustration of the RV reconstruction framework using BOLD signals and head motion parameters. The input to the model consists of 630 mean BOLD signals from functional ROIs and 6 head motion parameters (translations and rotations along the x, y, and z axes). Model inputs are constructed using a sliding window spanning a fixed temporal duration of 50 s, with the number of samples determined by the dataset-specific TR. Each window is used to reconstruct a single RV time point based on three temporal configurations: (1) future-only input, (2) combined past and future input, and (3) past-only input. The model output (orange) represents the reconstructed RV and is compared with the target signal (black), defined as the standard deviation of the respiratory waveform within a 6-s window centered at each time point.

To enable continuous RV reconstruction across the entire fMRI scan, we employed a sliding window approach defined over a fixed temporal duration (50 s). The number of samples per window was adjusted according to the repetition time (TR) of each dataset, ensuring that the model consistently captured comparable physiological context across acquisition protocols. Each window was mapped to a single output time point. Consecutive windows were generated using a stride of one time point (i.e., one TR), resulting in overlapping windows and enabling continuous reconstruction across the full scan duration. This approach, which we previously introduced in pediatric and adult cohorts (Addeh et al., 2023; Addeh, Vega, et al., 2025), uses three distinct temporal configurations to ensure full coverage of the RV time series. In the first configuration, the output corresponds to the first time point of the window and relies only on future information; in the second, the output corresponds to the center of the window and incorporates both past and future information; and in the third, the output corresponds to the last time point and relies only on past information. These configurations are illustrated in Figure 1. For each time point, the model input includes [50/TRs × 636] features, comprising 630 mean BOLD signals and 6 head motion traces over 65 TRs.

To ensure complete coverage of the RV waveform from beginning to end of the scan, the three temporal configurations were applied in a time-consistent manner. Specifically, Method 1 was used for time points within the first 25 s of the scan, Method 3 for time points within the final 25 s, and Method 2 for all intermediate time points. Method 2 covers the majority of the scan and is expected to yield higher performance due to its access to both past and future BOLD information. The RV target was computed from the recorded respiratory belt signal and defined as the standard deviation of the respiration waveform within a 6-s sliding window centered at each time point (Chang et al., 2009). This measured physiological signal served as the ground truth target for supervised training of the RV reconstruction model.

The choice of a 50-s window is motivated by the temporal characteristics of respiratory influences on the BOLD signal. Respiratory-driven CO_2_ fluctuations modulate cerebral blood flow and BOLD contrast with delays of approximately 10–20 s, and breathing cycles themselves evolve smoothly over tens of seconds. A 50-s window, therefore, provides sufficient temporal context for all three estimation configurations to capture both the delayed hemodynamic effects and the history-dependent structure of respiration. There is no strict physiological rule requiring exactly this midpoint; rather, our intention was to allow Method 2 to use both past and future BOLD information whenever both are available. Method 1 benefits from the full 50 s of preceding BOLD information, while Method 2 leverages 25 s of both past and future data, which contributes to its superior performance and motivates applying it over the majority of the scan. At the same time, the window is kept as short as physiologically justified to preserve temporal resolution, limit edge effects, and maintain computational efficiency. This balance makes the 50-s window an effective and robust choice for RV reconstruction across datasets and age groups.

#### 2.2.2 HRV reconstruction

HRV reconstruction is performed using the same 1D-CNN + GRU architecture as in RV estimation, with two key differences: head motion parameters are excluded from the input, and the reconstruction strategy is fixed to a single configuration. Specifically, for each 50-s input window, the model predicts the HRV value corresponding to the time point located 5 s from the start of the window, thereby leveraging both past and future information. Consecutive windows were generated using a stride of one time point (i.e., one TR), resulting in overlapping windows and enabling continuous HRV reconstruction across the scan.

**Fig. 2.**
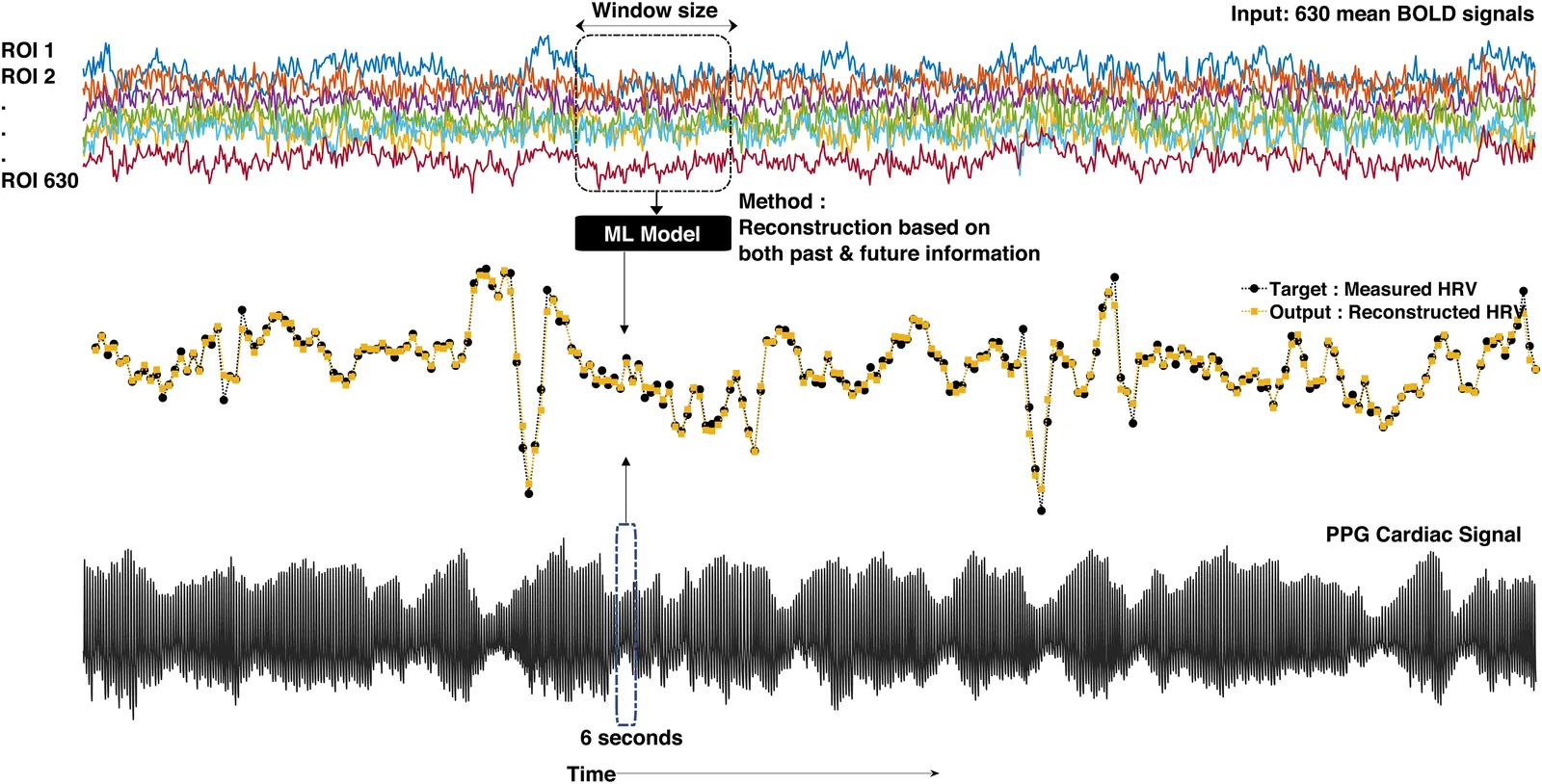
Illustration of the HRV reconstruction framework using BOLD signals. The model input consists of 630 mean BOLD signals from functional ROIs, segmented into sliding windows spanning a fixed temporal duration of 50 s, with the number of samples determined by the dataset-specific TR. For each window, the model predicts the HRV value at a fixed temporal offset (5 s) within the window, enabling the use of both past and future contextual information. The reconstructed HRV output (orange) is compared against the ground-truth target (black), defined as the inverse of the mean inter-beat interval computed from the PPG signal within a 6-s sliding window.

The target HRV was derived from the measured PPG signal and defined as the inverse of the mean inter-beat interval (IBI) within a 6-s sliding window centered at each time point (Chang et al., 2009). This measured physiological signal served as the ground truth for supervised training (Chang et al., 2009). This approach is illustrated in Figure 2. Due to the windowing configuration, HRV values cannot be estimated for the first 5 s and the last 45 s of each scan.

Heart rate influences on the BOLD signal exhibit both short-latency effects (approximately 6–12 s) and longer-latency components (approximately 30–42 s), and HRV itself reflects the cumulative history of preceding cardiac cycles. Positioning the prediction 5 s into each 50-s window enables the model to incorporate the minimal past information needed to represent recent cardiac history while maximizing access to future BOLD samples, where the delayed cardiac-related effects appear most strongly. This placement balances physiological relevance with practical constraints: using the midpoint of the window, as done for RV, would require substantially longer windows (e.g., ~80 s) to capture both short- and long-delay cardiac effects symmetrically, which would in turn create large edge-loss regions at the beginning and end of each scan. By keeping the window as short as physiologically justified and placing the prediction near the beginning of the window, the method minimizes edge effects while retaining the temporal information necessary for accurate HRV reconstruction.

### 2.3 Network architecture

The proposed architecture integrates 1D-CNNs and GRUs to reconstruct RV and HRV directly from resting-state BOLD-fMRI signals. The 1D-CNN layers extract short-range temporal features within each signal stream, effectively capturing localized physiological fluctuations such as abrupt changes associated with respiration or cardiac cycles. These features are subsequently passed to GRU layers, which model longer-term temporal dependencies across the 50 s input window. This hybrid design allows the model to learn transient and sustained patterns essential for understanding the physiological dynamics embedded in the BOLD signal.

Recognizing the variability in physiological properties, motion artifacts, and BOLD signal characteristics across the lifespan, we implemented cohort-specific models for HCP-D, HCP-YA, and HCP-A. For each group, the model architecture was customized by adjusting key hyperparameters, including the number of convolutional filters, kernel sizes, GRU units, and dropout rates, guided by prior neurophysiological literature describing age-related differences in motion characteristics, physiological variability, and BOLD signal dynamics (Dosenbach et al., 2010; Fair et al., 2020; Kaplan et al., 2022), together with practical considerations related to cohort-specific signal characteristics. These hyperparameters were selected a priori and held fixed during training, without exhaustive grid search, to reduce overfitting risk. Early stopping based on validation performance was used during training to further control overfitting under cohort-specific conditions.

This age-specific architectural adaptation ensures that the model accommodates developmental and aging-related differences in neurovascular coupling, motion behavior, and physiological confound patterns. A full description of the architecture and selected hyperparameters for each cohort, along with a schematic illustration of the network, is provided in Supplementary Materials, Section 2.

### 2.4 Model training

To account for developmental and physiological variability across the lifespan, separate machine learning models were trained for each age group using data exclusively from the corresponding HCP cohort. Specifically, the model for RV and HRV estimation in the HCP-D cohort was trained using only HCP-D data, while models for HCP-YA and HCP-A were similarly trained on their respective datasets. This cohort-specific training strategy enabled each model to learn age-relevant BOLD signal characteristics and physiological dynamics. Model tuning, training, and evaluation were performed independently within each cohort, and no model was trained on one age group and applied to another.

Model development was restricted to participants with high-quality physiological recordings, including belt-derived respiratory traces and PPG-based cardiac signals. Quality control procedures involved artifact removal, motion-based scan exclusion, and physiological trace validation (see Supplementary Materials, Section 3). After applying these criteria, 550 resting-state fMRI scans from 411 participants (out of 2,451 total scans) were selected from HCP-D, 1050 scans from 590 individuals (out of 3,946 scans) were selected from HCP-YA, and 700 scans from 429 participants (out of 2,187 scans) were selected from HCP-A. All models were trained using the mean squared error (MSE) loss function.

To evaluate our model, we employed a 10-fold cross-validation methodology, consistent with standard practices (Bishop & Nasrabadi, 2006; Kuhn & Johnson, 2013). The input–output dataset was divided into 10 subsets. For each cross-validation fold, nine subsets were used for training the model, while the remaining subset was designated for testing. This process was repeated 10 times, each time with a different subset as the test set, ensuring that each subset was utilized exactly once for testing. Additionally, 20% of the data within the training segment was allocated to validation in each fold (see Supplementary Materials, Section 4). To prevent data leakage, data partitioning was performed at the subject level rather than the scan level, such that all scans from a given participant were assigned exclusively to either the training, validation, or test set within each fold. Each model was trained for 300 epochs per fold. Here, an epoch denotes one complete pass through the training data in that fold using mini-batch optimization, with 300 epochs representing an upper bound on training duration. This systematic rotation of subsets across folds ensures robust model evaluation by maintaining independent training and testing subsets across all iterations, minimizing potential biases.

To evaluate the model’s performance post-training, we used MAE, MSE, r, and DTW (for more details, see Supplementary Materials, Section 5). Physiological fluctuations are known to influence the BOLD signal with characteristic hemodynamic delays, with respiratory-related effects peaking on the order of 10–20 s and heart rate-related effects spanning both shorter and longer latencies. In the present work, these delays were not explicitly modeled or corrected during training or evaluation. Instead, they were handled implicitly through the use of temporally extended input windows that provide the model with access to temporally offset BOLD samples relative to each target time point (Bayrak et al., 2025). The objective of the model is to reconstruct underlying physiological waveforms (RV and HRV) rather than to model the forward hemodynamic response. Accordingly, no post-hoc temporal shifting or alignment was applied between reconstructed and measured RV/HRV signals. All evaluation metrics (MAE, MSE, Pearson correlation, and DTW) were computed directly between reconstructed and measured physiological time series defined on the same fMRI temporal grid, with DTW included to account for potential local timing variability without imposing a fixed temporal lag.

Performance metrics from all iterations were compiled, and results are reported as the “Average ± Standard Error (SE)” to clearly indicate the model’s overall efficacy. To effectively demonstrate the method’s performance, violin plots were used, illustrating the model’s performance over the unseen test data throughout the 10-fold cross-validation process. Additional technical details are provided in Supplementary Materials, Section 6.

### 2.5 Functional connectivity analyses

To assess the impact of RV and HRV reconstruction on subsequent fMRI analyses, we examined changes in resting-state functional connectivity (FC) following physiological correction. Functional connectivity analyses were restricted to the 518 cortical ROIs of the D-BFA atlas, as cortical regions provide the most interpretable and neurally relevant correlations for resting-state FC, while subcortical, white matter, and CSF regions were excluded to avoid contamination by non-neuronal physiological fluctuations.

Physiological effects were removed using a general linear model (GLM) framework, with regression coefficients estimated via ordinary least squares. To explicitly account for the hemodynamic delay between physiological fluctuations and the BOLD signal, measured or reconstructed RV and HRV signals were first convolved with canonical physiological response functions. Specifically, RV was convolved with a respiration response function (RRF) (Birn et al., 2008), and HRV was convolved with a cardiac response function (CRF) as described by Chang et al. (2009). Temporal and dispersion derivatives of each convolved regressor were additionally included to accommodate variability in response timing and shape (Chen et al., 2020). The resulting physiological basis set, together with rigid-body head-motion parameters and their temporal derivatives, was regressed voxel wise from the BOLD time series. Residual voxel time series were then averaged within each of the 518 cortical ROIs, and functional connectivity was computed as pairwise Pearson correlations between the resulting ROI-level residual time series.

Aside from motion correction and spatial normalization performed during initial preprocessing, no additional spatial smoothing or temporal band-pass filtering was applied prior to functional connectivity estimation. This choice was made to preserve physiologically meaningful variance and to ensure that observed FC changes primarily reflected the effects of physiological regression rather than preprocessing choices. No additional nuisance regressors beyond rigid-body motion parameters and RV/HRV regressors were included.

To evaluate whether reconstructed physiological regressors yield FC corrections comparable with those obtained using measured physiology, we quantified agreement between FC matrices corrected using measured versus estimated RV/HRV, following prior work demonstrating that predicted physiological signals can induce connectivity changes comparable with those obtained using recorded physiology (Bayrak et al., 2025). For each subject, similarity was assessed by correlating the upper-triangular elements of the two ROI-ROI FC matrices (“matrix similarity”). In addition, to determine whether reconstructed RV/HRV removes the same connectivity component as measured RV/HRV, we computed correction-induced changes relative to baseline FC (∆FC = FC_corrected_ – FC_baseline_). Baseline FC was computed using the same processing pipeline but without RV/HRV regressors, and ∆FC similarity was quantified by correlating the upper-triangular elements of the resulting matrices. Distributions of subject-wise FC and ∆FC similarity metrics were used to summarize agreement across the cohort.

### 2.6 Evaluation of spatial parcellation and model effects

To evaluate the influence of spatial representation and modeling strategy on physiological signal reconstruction, we systematically compared multiple ROI parcellations and machine learning architectures under a unified input framework. All models were trained and tested using identical preprocessing, windowing schemes, and evaluation metrics to ensure fair and consistent comparison.

With respect to spatial representation, 2 commonly used static parcellations were included alongside the dynamic functional atlas: the Glasser multi-modal cortical atlas, comprising 360 functionally defined cortical ROIs (Glasser et al., 2016), and the Harvard–Oxford anatomical atlas, comprising 69 structurally defined ROIs (Makris et al., 2006). These parcellations were used to assess how different spatial definitions affect RV and HRV reconstruction performance. The structurally defined atlas was included solely as a baseline comparator to assess the effect of anatomical versus functional parcellation on physiological signal reconstruction, whereas anatomically defined white matter and CSF ROIs were incorporated across configurations to capture tissue-specific physiological fluctuations rather than to represent a competing structural parcellation.

### 2.7 Network-level contribution analysis

To further characterize how different brain systems contribute to physiological signal reconstruction within the optimal input configuration, we performed a network-level contribution analysis, including default mode, salience, executive control, sensorimotor, visual, visuospatial, auditory, language, and basal ganglia networks.

Network contributions were evaluated using a perturbation-based ablation approach. Specifically, for each functional network, ROIs belonging to that network were excluded from the model input, while all remaining cortical, subcortical, WM, and CSF ROIs were retained. Model training and evaluation were then repeated using the same cross-validation folds, architecture, preprocessing pipeline, and training protocol as the full configuration to ensure comparability.

The impact of each network was quantified as the change in reconstruction performance relative to the full model. For clarity and interpretability, network-level effects are summarized using changes in the Pearson correlation coefficient (r), which provides a scale-invariant measure of temporal agreement between reconstructed and reference physiological signals.

### 2.8 Architectural comparison and temporal window sensitivity analysis

To assess the impact of machine learning architecture on physiological signal reconstruction, we performed a systematic comparison of multiple modeling strategies under matched input and training conditions. Specifically, the proposed 1D-CNN + GRU architecture was evaluated against three established baselines: a previously published 1D-CNN model (Salas et al., 2021), our earlier 1D-CNN framework (Addeh, Vega, et al., 2025), and a sequence-to-sequence bi-LSTM model (Bayrak et al., 2025). All models were trained and evaluated using identical preprocessing, ROI time series, and native temporal resolution (TR = 0.72 s). For window-based architectures, a fixed temporal context of 50 s was used for the primary architectural comparison, whereas the bi-LSTM model operated on the full fMRI time series. Reconstruction performance across architectures was evaluated using MAE, MSE, r, and DTW.

To further disentangle architectural temporal modeling capacity from the effect of input window length, we conducted an additional sensitivity analysis in which the temporal window size was systematically varied from 30 to 100 s for all window-based models. In this analysis, DTW was used to quantify temporal alignment and sensitivity to local timing variability that may not be fully captured by correlation-based measures. This approach was designed to assess whether observed performance differences were attributable primarily to increased temporal context length or to differences in architectural temporal modeling capacity. For both experiments, statistical comparisons across model configurations were performed using the Friedman test.

## 3 Results

### 3.1 Performance of the proposed method on the young adult cohort

#### 3.1.1 Model evaluation

In this experiment, we evaluated the proposed deep learning model for reconstructing RV and HRV using resting-state fMRI data from the HCP-YA dataset. This dataset was selected due to its superior quality in both fMRI and physiological recordings, attributed to participant compliance and optimized acquisition protocols compared with HCP-D and HCP-A.

**Fig. 3.**
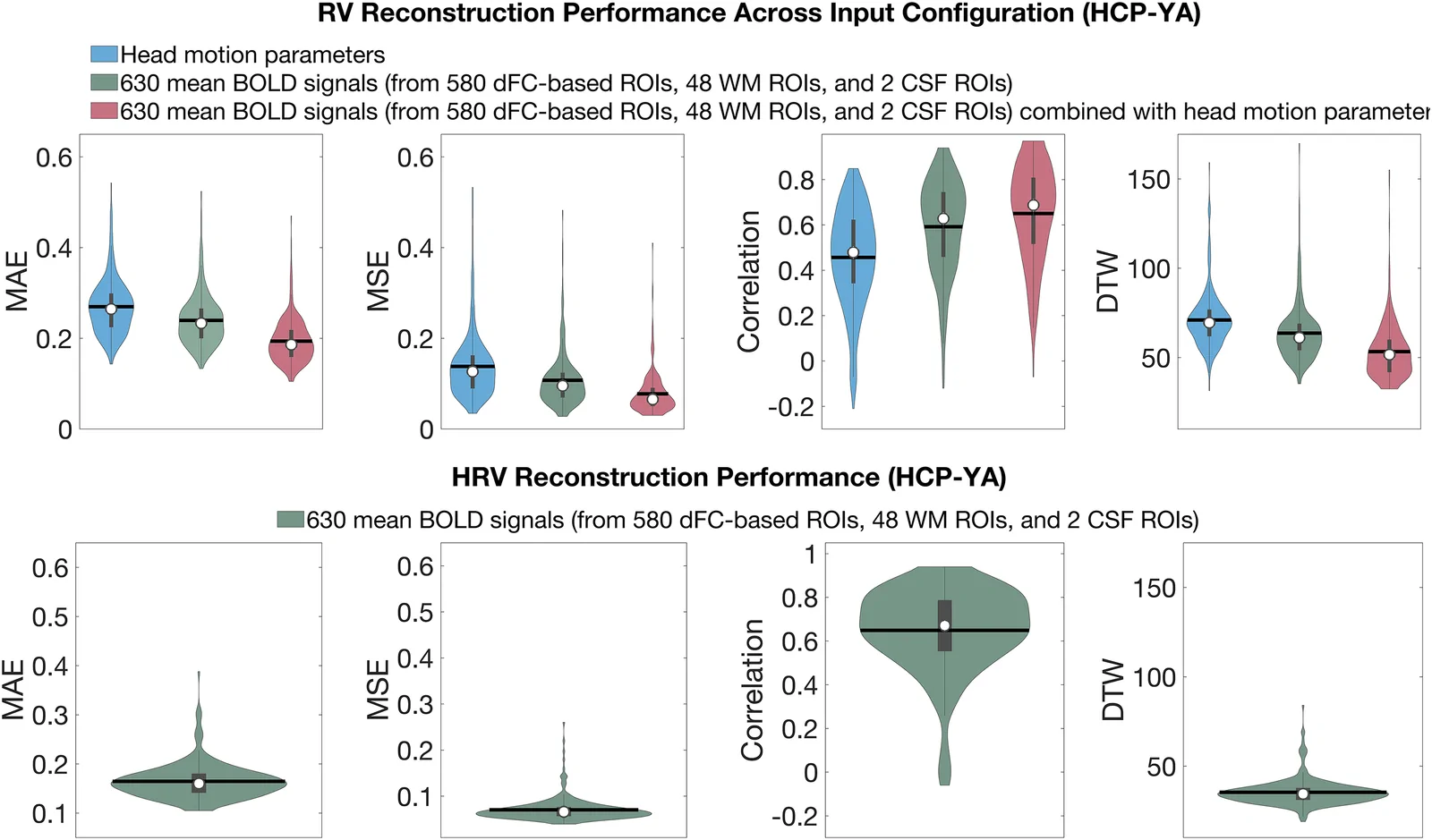
Effect of input configuration on RV and HRV reconstruction performance (HCP-YA). Violin plots illustrate the distribution of evaluation metrics, including mean absolute error (MAE), mean squared error (MSE), Pearson correlation coefficient (r), and dynamic time warping distance (DTW), across the test set. Top row: RV reconstruction performance for 3 input configurations: head motion parameters only (blue), mean BOLD signals from 630 ROIs (green), and a combined input of BOLD signals and head motion parameters (red). The combined configuration consistently achieves the best performance across all metrics, indicating that motion parameters provide complementary information when integrated with spatially enriched BOLD signals. Bottom row: HRV reconstruction performance using mean BOLD signals from 630 ROIs. Results demonstrate robust HRV estimation across metrics, supporting the ability of spatially distributed BOLD signals to capture low-frequency cardiac-related fluctuations.

To assess model performance for RV reconstruction, we trained and tested the network using 3 input configurations: (1) head motion parameters alone, (2) mean BOLD signals from 630 predefined brain ROIs (comprising 580 gray matter ROIs, including 518 cortical and 62 subcortical regions, 48 white matter ROIs, and 2 CSF ROIs), and (3) a combined input of BOLD signals and head motion parameters. For HRV reconstruction, model evaluation was performed using mean BOLD signals from the same set of 630 ROIs, as head motion parameters were not included for HRV estimation. The model performance for RV and HRV reconstruction is shown in Figure 3. The model achieved the highest accuracy when both BOLD signals and motion parameters were used as input. This configuration provided stronger temporal alignment with the measured RV traces and yielded lower reconstruction error across all evaluation metrics. A Friedman test confirmed that input configuration had a statistically significant effect on RV estimation accuracy (p < 0.001).

For HRV reconstruction, the inclusion of head motion parameters did not improve performance and, in some cases, slightly degraded it. Therefore, head motion parameters were excluded from the HRV reconstruction model. Representative examples of RV and HRV waveform reconstructions for individual subjects are provided in the Supplementary Materials (Section 7).

#### 3.1.2 Impact of ROI selection on model performance

To evaluate the influence of spatial input configuration on model performance, we tested four different ROI-based configurations using the HCP-YA dataset:

- Configuration 1: 69 Structural ROIs
- Configuration 2: 580 dFC-based ROIs
- Configuration 3: 360 Multi-Modal Cortical ROIs + 48 WM ROIs + 2 CSF ROIs
- Configuration 4: 580 dFC-based ROIs + 48 WM ROIs + 2 CSF ROIs

**Fig. 4.**
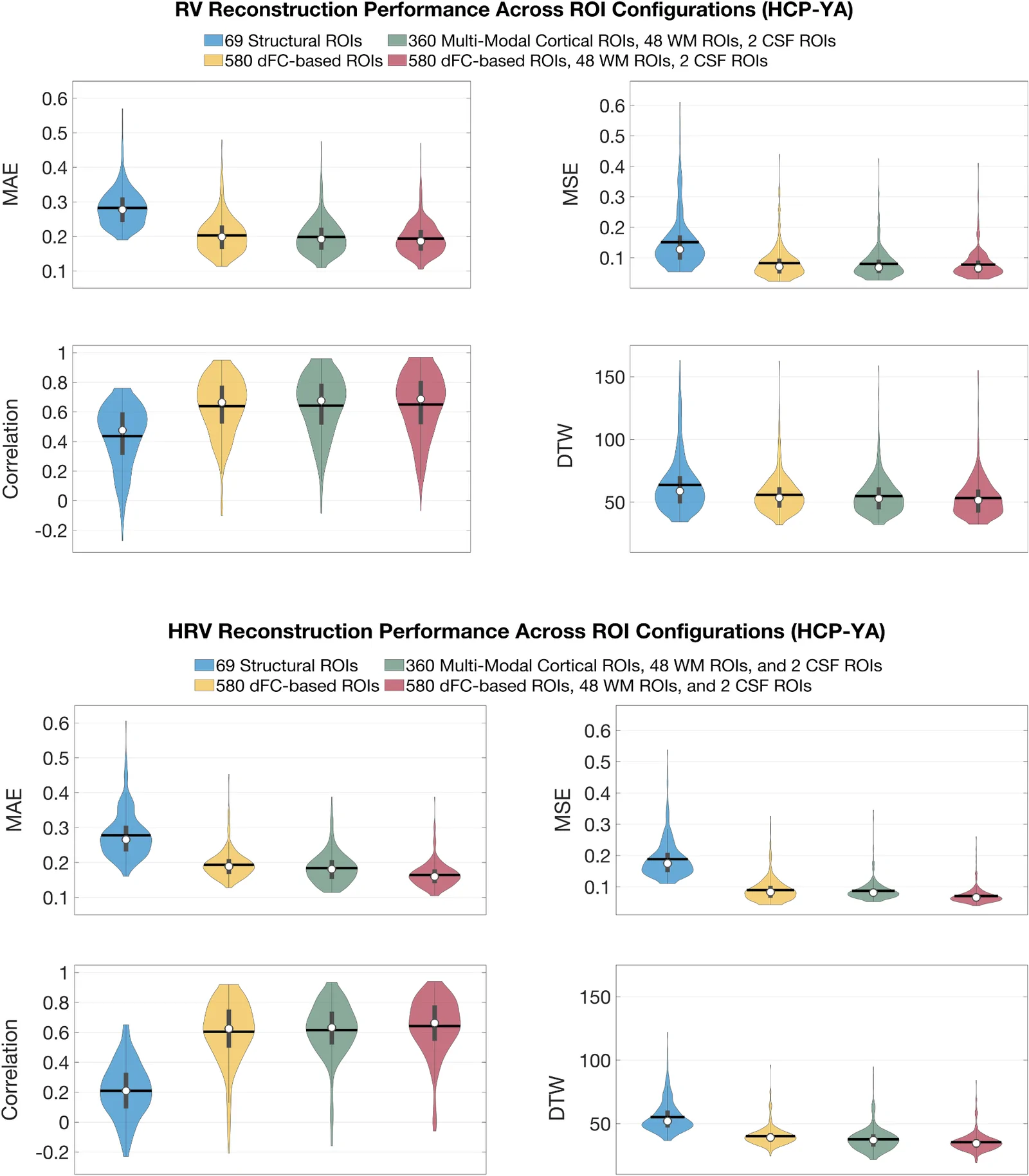
RV and HRV reconstruction performance across different ROI configurations using the HCP-YA dataset. Violin plots display the distribution of evaluation metrics, including MAE, MSE, Pearson correlation coefficient (r), and DTW, for RV and HRV reconstruction under 4 input configurations: 69 structural ROIs (blue), 580 dFC-based ROIs (orange), 360 multi-modal cortical ROIs with 48 WM and 2 CSF ROIs (green), and 580 dFC-based ROIs with 48 WM and 2 CSF ROIs (red). Among the tested configurations, the combination of dFC-based ROIs with white matter and CSF regions (red) achieved the best overall performance across all metrics, indicating the benefit of incorporating both dynamic functional and non-gray matter inputs in the model.

For RV reconstruction, six head motion parameters were appended to the BOLD signal inputs in all configurations. Figure 4 illustrates the impact of ROI selection on RV and HRV reconstruction performance, respectively.

**Fig. 5.**
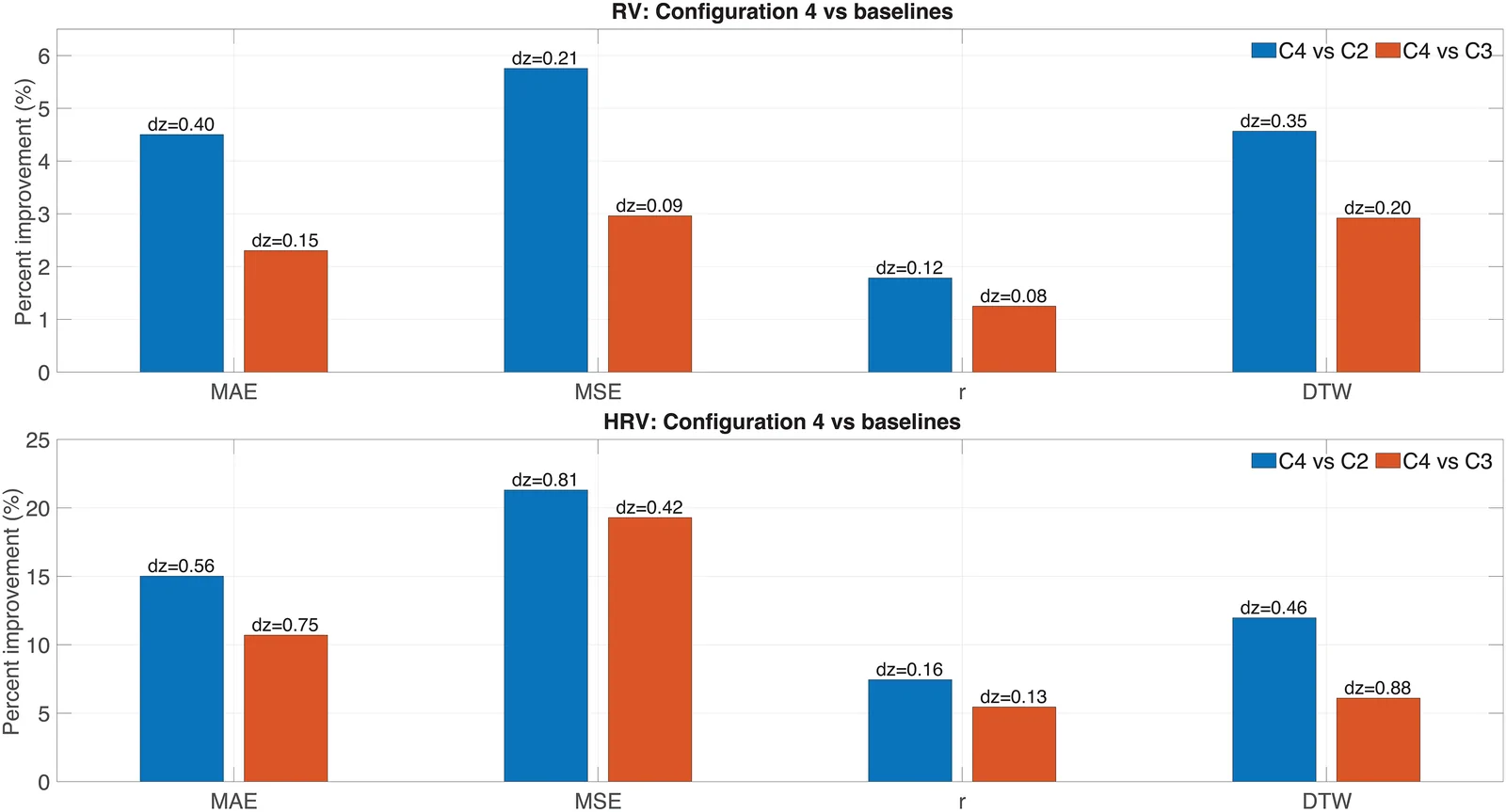
Percent improvement of Configuration 4 relative to baseline configurations for RV and HRV reconstruction. The figure summarizes the relative percent improvement of Configuration 4 (C4: 580 dFC-based ROIs + 48 white matter (WM) ROIs + 2 cerebrospinal fluid (CSF) ROIs) compared with 2 baselines: Configuration 2 (C2: 580 dFC-based ROIs) and Configuration 3 (C3: 360 multi-modal cortical ROIs + 48 WM ROIs + 2 CSF ROIs). The top panel shows results for respiratory variation (RV) reconstruction, and the bottom panel shows results for heart rate variation (HRV) reconstruction. Bars indicate the mean percent improvement of C4 relative to each baseline across four evaluation metrics (MAE, MSE, Pearson correlation coefficient r, and DTW). Values annotated above each bar report Cohen’s dz, quantifying the standardized effect size of the paired comparison. Overall, RV reconstruction exhibits modest improvements with small-to-moderate effect sizes, whereas HRV reconstruction demonstrates substantially larger improvements and effect sizes across all metrics, highlighting the stronger sensitivity of HRV estimation to both expanded spatial coverage and dynamic functional parcellation.

For RV reconstruction, the Friedman test revealed a significant overall effect of ROI configuration across all evaluation metrics (MAE, MSE, r, and DTW; all p < 0.001), indicating that model performance depended on the spatial input representation. To further quantify the magnitude of these differences, Figure 5 summarizes the relative percent improvement of Configuration 4 over Configurations 2 and 3, together with corresponding effect sizes.

Effect sizes are reported as Cohen’s dz, reflecting standardized paired-sample differences. Pairwise Wilcoxon signed-rank tests showed that Configuration 4 (580 dFC-based ROIs with additional WM and CSF regions) consistently outperformed Configuration 2 (580 dFC-based ROIs alone) for error-based metrics. Specifically, Configuration 4 yielded significantly lower MAE (p = 0.0017, dz = 0.40) and DTW (p = 0.0031, dz = 0.35), corresponding to mean improvements of approximately 4–6%. Improvements in MSE were smaller (p = 0.032, dz = 0.21), while differences in correlation (r) did not reach statistical significance. Comparisons between Configuration 4 and Configuration 3 (multi-modal cortical ROIs with WM and CSF) showed similar trends but did not survive significance testing, with small effect sizes across all metrics. Overall, RV reconstruction benefited modestly from the inclusion of WM and CSF regions when combined with dFC-based ROIs, with the strongest gains observed for temporal alignment and absolute error metrics.

In contrast to RV, HRV reconstruction showed a markedly stronger dependence on ROI configuration, as reflected in both the distributional results (Fig. 4) and the substantially larger percent improvements and effect sizes (Fig. 5). Friedman tests demonstrated highly significant effects of configuration across all metrics (all p < 0.001). Pairwise comparisons revealed that Configuration 4 significantly outperformed both Configuration 2 and Configuration 3 across all evaluation measures. Relative to Configuration 2, Configuration 4 achieved substantial reductions in MAE (p < 0.001, dz = 0.56), MSE (p < 0.001, dz = 0.81), and DTW (p < 0.001, dz = 0.46), alongside improved correlation (p = 0.0035, dz = 0.16). Similar or larger effects were observed relative to Configuration 3, with effect sizes reaching the large range for DTW (dz = 0.88) and MAE (dz = 0.75). Mean percentage improvements for HRV reconstruction ranged from approximately 6 to 21%, depending on the metric and baseline configuration. These results indicate that HRV reconstruction benefits strongly from both expanded spatial coverage and the use of dynamic functional parcellations, with Configuration 4 providing the most informative input representation across all metrics.

#### 3.1.3 Network-level contributions to RV and HRV reconstruction

**Fig. 6.**
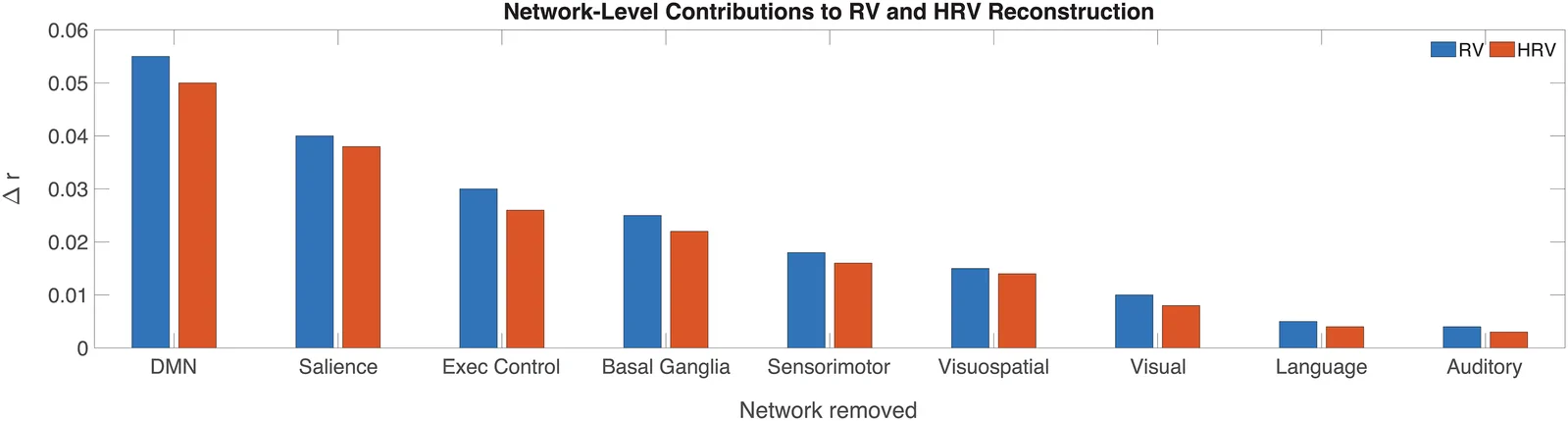
Network-level contributions to RV and HRV reconstruction assessed using a perturbation-based ablation approach. Bars show the change in Pearson correlation coefficient (∆r) following removal of individual functional networks, while white matter and cerebrospinal fluid regions were retained in all configurations. Larger ∆r values indicate greater sensitivity of reconstruction performance to removal of the corresponding network.

Figure 6 summarizes the network-level contribution analysis for RV and HRV reconstruction using a perturbation-based ablation approach. Removal of individual functional networks resulted in modest but consistent reductions in reconstruction accuracy, indicating that performance is supported by distributed contributions across multiple brain systems, rather than being driven by a single network.

For both RV and HRV, the largest decrease in performance was observed following removal of the DMN (∆r ~ 0.055 for RV; ∆r ~ 0.050 for HRV), followed by the salience network (∆r ~ 0.040 for RV; ∆r ~ 0.038 for HRV). The executive control and basal ganglia networks showed moderate contributions, with intermediate reductions in correlation when ablated. In contrast, sensorimotor, visuospatial, and visual networks produced smaller but non-negligible effects, while language and auditory networks had minimal impact on reconstruction performance.

Across all networks, RV reconstruction exhibited slightly greater sensitivity to network removal than HRV, although the overall pattern of network contributions was highly consistent between the two signals. Importantly, no individual network ablation resulted in a large reduction in reconstruction performance for either RV or HRV.

### 3.2 Performance in developmental and aging cohorts

To evaluate the performance of the proposed framework across different age groups, we assessed its reconstruction accuracy separately in the HCP-D and HCP-A cohorts. Importantly, the architecture and hyperparameters of the proposed 1D-CNN + GRU model were optimized independently for each group to accommodate age-related variations in BOLD signal characteristics, motion profiles, and physiological confound patterns.

**Fig. 7.**
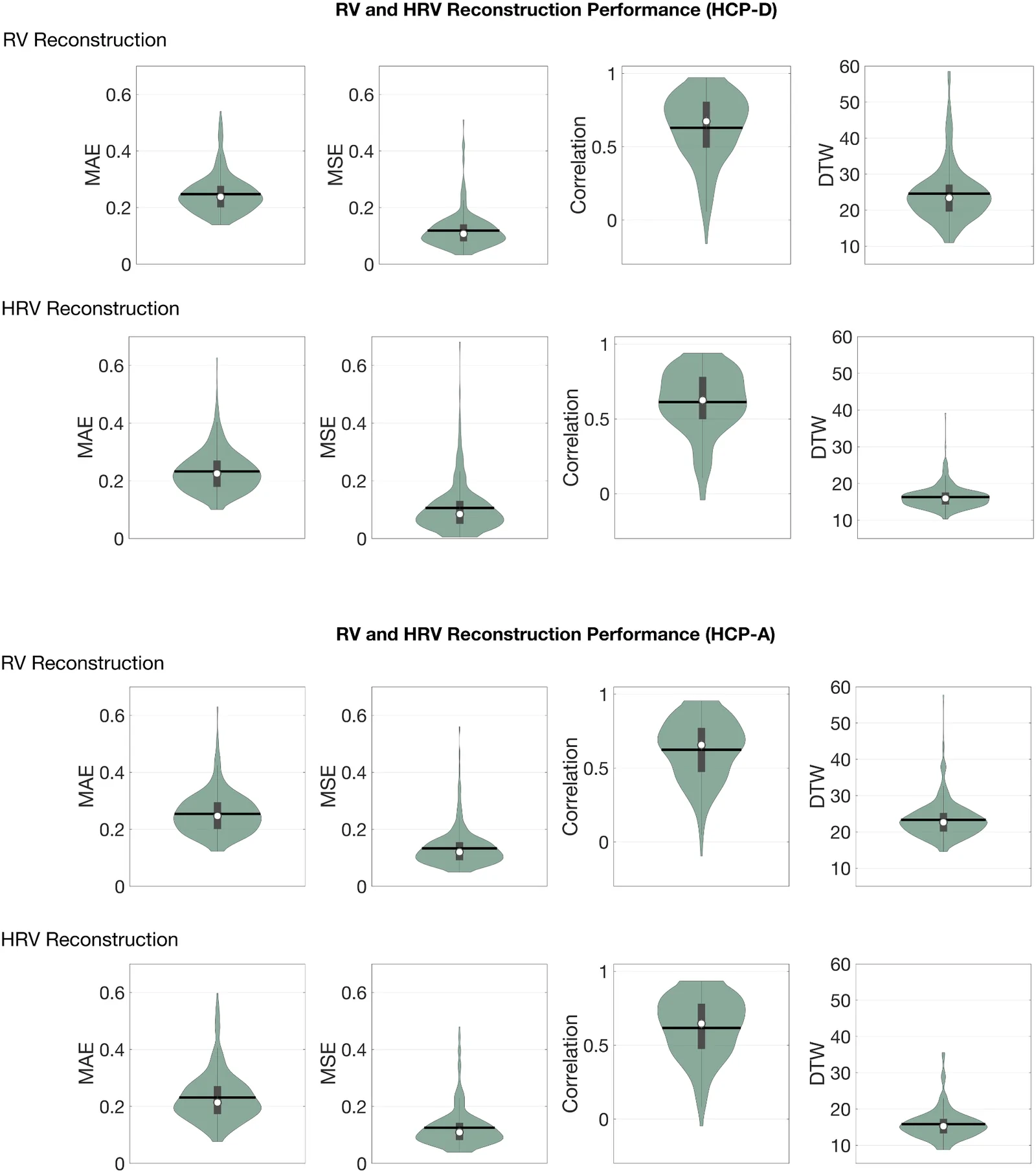
Reconstruction performance of RV and HRV on the HCP-D and HCP-A cohorts. Violin plots summarize RV and HRV reconstruction performance across test subjects using the proposed model. Metrics include MAE, MSE, Pearson’s r, and DTW. The model achieved consistent performance in the developmental and aging populations, indicating effective generalization across the lifespan.

Figure 7 presents the RV and HRV reconstruction results for the developmental and aging cohorts, respectively. In both age groups, the model demonstrated consistent and reliable performance across all evaluation metrics (MAE, MSE, Pearson’s r, and DTW), closely matching the trends observed in the young adult group. This consistency highlights the robustness of the proposed approach across distinct neurodevelopmental and neurodegenerative stages.

These results indicate that, with appropriate model tuning, the proposed architecture can effectively capture physiologically driven signal fluctuations in BOLD data across the human lifespan. The strong performance across all age cohorts supports the feasibility of applying this framework to pediatric and aging populations, where physiological variability is typically greater and traditional correction methods may be less effective.

### 3.3 Functional connectivity analyses

**Fig. 8.**
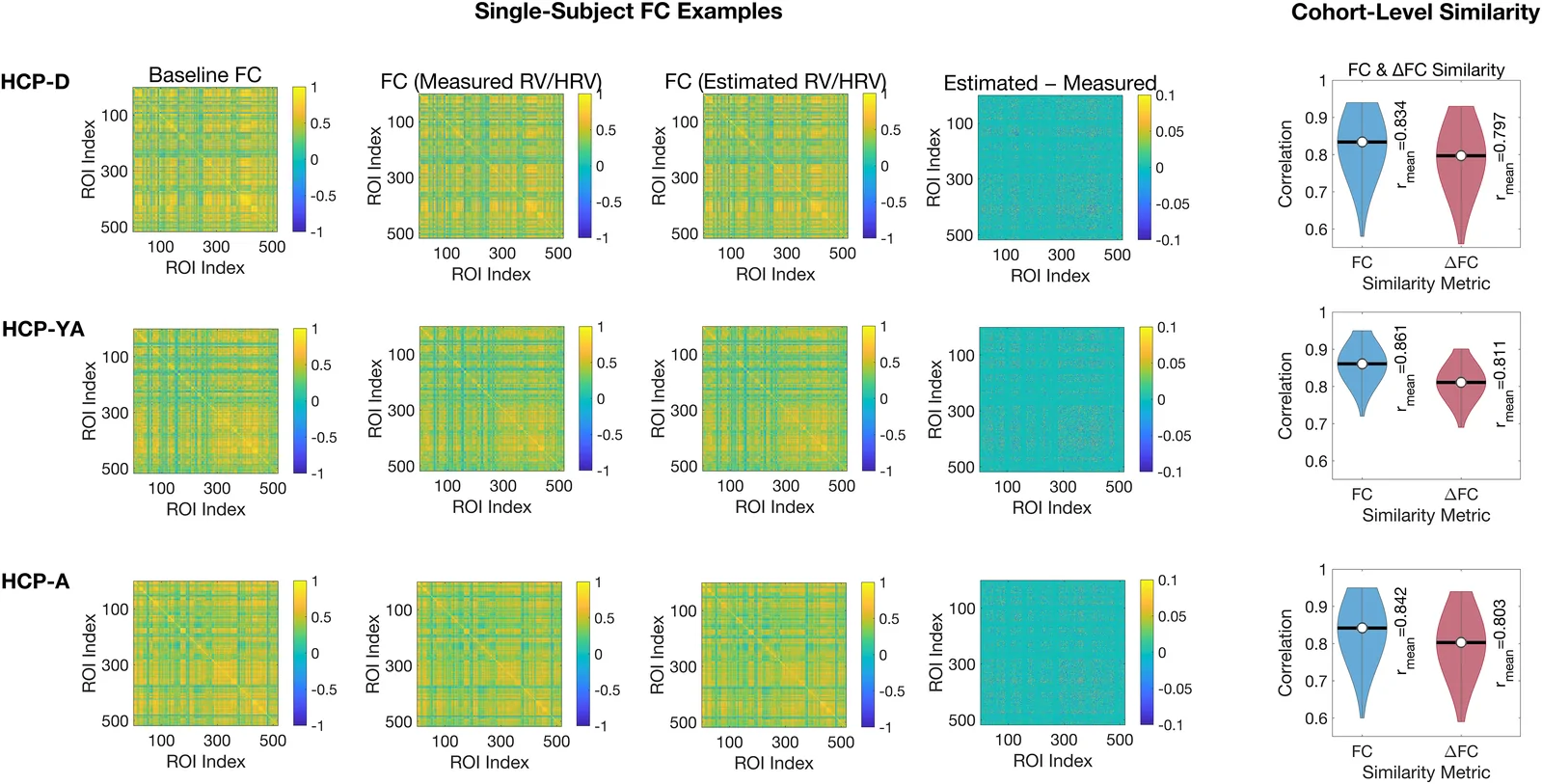
Impact of reconstructed RV/HRV on resting-state functional connectivity across the lifespan. Columns 1–4 show representative single-subject FC matrices from the HCP-D (top), HCP-YA (middle), and HCP-A (bottom) cohorts, including baseline FC (no physiological correction), FC corrected using measured RV/HRV, FC corrected using reconstructed RV/HRV, and the difference between estimated- and measured-corrected FC. Column 5 summarizes cohort-level agreement between measured- and estimated-corrected FC using similarity metrics computed across subjects. Violin plots show distributions of FC matrix similarity and correction-induced ∆FC similarity, quantified as correlations between the upper-triangular elements of the corresponding FC matrices. Mean similarity values are indicated for each cohort.

To assess the downstream impact of RV–HRV reconstruction on resting-state functional connectivity, we compared FC estimates obtained after physiological correction using measured versus reconstructed RV/HRV signals across the HCP-D, HCP-YA, and HCP-A cohorts. Figure 8 summarizes both representative single-subject FC matrices and cohort-level similarity metrics.

Across all cohorts, FC matrices corrected using reconstructed RV/HRV closely resembled those obtained using measured physiological recordings. Representative single-subject examples (Fig. 8, columns 1–3) demonstrate that large-scale FC structure was preserved following physiological correction, regardless of whether measured or reconstructed RV/HRV regressors were used. Difference maps between estimated- and measured-corrected FC (Fig. 8, column 4) exhibited small-magnitude, spatially diffuse residuals centered near zero, indicating minimal systematic differences between the two correction approaches.

Across the cohort, FC matrix similarity between measured- and estimated-corrected FC was high in all datasets (Fig. 8, column 5), with mean correlation coefficients of approximately 0.83 in HCP-D, 0.86 in HCP-YA, and 0.84 in HCP-A. These results indicate that reconstructed RV/HRV regressors yield FC estimates that are highly consistent with those obtained using recorded physiology.

To further evaluate whether reconstructed RV/HRV removed the same connectivity component as measured RV/HRV, we examined correction-induced changes relative to baseline FC (∆FC). ∆FC similarity was also high across cohorts, with mean values of approximately 0.80 in HCP-D, 0.81 in HCP-YA, and 0.80 in HCP-A.

### 3.4 Impact of architecture choice within cohorts

To isolate the effect of architectural design independently of training data and cohort-specific signal properties, we performed a within-cohort architecture comparison using the three age-specific network configurations defined in Section 2. For each cohort, all architectures were trained and evaluated using data exclusively from that same cohort. Thus, when an architecture originally designed for a different age group was evaluated (e.g., an HCP-D–inspired architecture applied within the HCP-YA cohort), it was retrained and tested entirely on HCP-YA data. This controlled design ensures that any observed performance differences reflect intrinsic architectural suitability rather than differences in dataset composition, training data availability, or cross-cohort transfer effects.

**Fig. 9.**
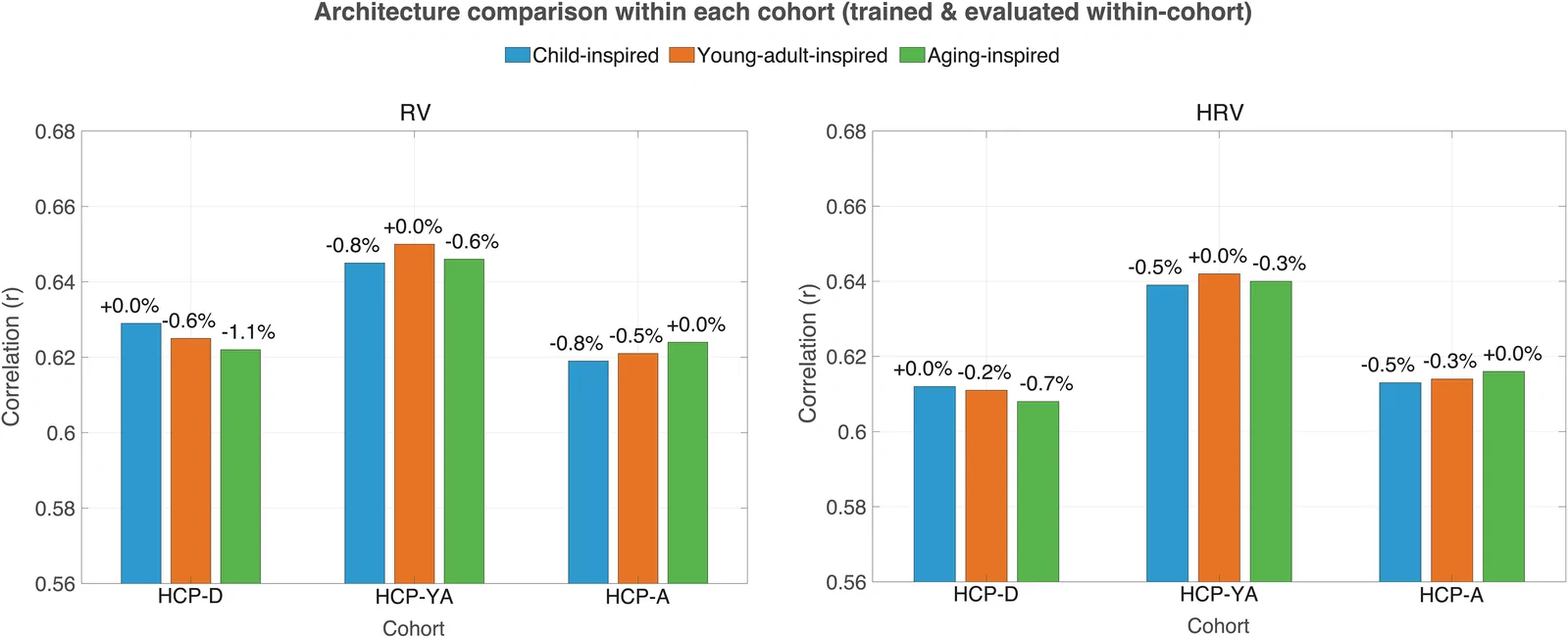
Impact of architecture choice within cohorts on RV and HRV reconstruction performance. Bar plots show Pearson correlation coefficients (r) for RV (left) and HRV (right) reconstruction across the HCP-D, HCP-YA, and HCP-A cohorts. For each cohort, three age-specific network architectures were evaluated: child-optimized, young-adult-optimized, and aging-optimized. All architectures were trained and evaluated within the same cohort, ensuring that differences reflect architectural design rather than training data or cross-cohort effects. Percentage values above bars indicate relative changes in correlation with respect to the within-cohort optimized architecture (baseline). Across all cohorts and for both RV and HRV, the cohort-optimized architecture achieved the highest correlation, while architectures designed for other age groups showed small but consistent performance reductions (typically < 1%), which were statistically significant (p < 0.01).

Figure 9 summarizes RV and HRV reconstruction performance across cohorts under the within-cohort architecture comparison using Pearson’s correlation (r) for clarity. For each cohort, the architecture specifically optimized for that age group consistently achieved the highest Pearson correlation and, therefore, serves as the within-cohort reference. Architectures originally designed for other age groups exhibited small but systematic reductions in performance, with decreases in correlation generally remaining below 1%. These differences were consistent across both RV and HRV and across all cohorts, and were statistically significant (Friedman test, p < 0.01), indicating that architectural design choices tuned to cohort-specific signal characteristics confer a modest yet reproducible performance advantage. Similar trends were observed for error- and alignment-based metrics (MAE, MSE, and DTW).

Collectively, these results demonstrate that the proposed 1D-CNN + GRU framework is robust to variations in architectural configuration, while also benefiting from age-specific architectural tuning. Because all models were trained and evaluated on identical cohort data, the observed performance differences can be attributed to intrinsic architectural suitability rather than differences in training data, sample size, or cross-cohort transfer. These findings underscore the importance of adapting network design to age-related differences in physiological dynamics and BOLD signal properties when aiming to maximize reconstruction accuracy.

### 3.5 Impact of machine learning architecture

To evaluate the influence of model architecture on reconstruction performance, we compared the proposed 1D-CNN + GRU framework against several established architectures, including a previously published 1D-CNN model by Salas et al. (2021), a CNN-based model from Addeh, Vega, et al. (2025), and a sequence-to-sequence bidirectional long short-term memory (bi-LSTM) model by Bayrak et al. (2025). For this comparison, we focused on the HCP-YA dataset, which has been used in prior studies evaluating these baseline models. All models were trained and evaluated using the native temporal resolution (TR = 0.72 s) without temporal downsampling and with matched input features. This choice is further supported by an analysis of the spectral content of measured RV and HRV signals, which reveals physiologically meaningful power beyond the conventional 0.15 Hz cutoff when analyzed at native temporal resolution (Supplementary Materials, Section 8). Accordingly, the proposed framework was designed to preserve a broader physiological frequency spectrum and address reconstruction of higher-frequency physiological fluctuations that may be attenuated by aggressive filtering or temporal downsampling.

For window-based models, a fixed temporal context of 50 s was used, selected to balance physiological relevance, temporal resolution, edge effects, and computational efficiency. The CNN- and GRU-based models operate on fixed-length temporal windows, whereas the bi-LSTM model follows the original DeepPhysioRecon implementation (Bayrak et al., 2025) and performs sequence-to-sequence reconstruction over the full fMRI time series. Transformer-based architectures were not included in this comparison, as their effective deployment generally entails higher computational cost and memory requirements than CNN- or RNN-based models, particularly when operating on high temporal resolution fMRI data.

As summarized in Table 1, the proposed 1D-CNN + GRU framework achieved the highest reconstruction accuracy across all evaluated architectures for both RV and HRV. Relative to CNN-only baselines, the proposed model yielded consistent improvements across all evaluation metrics. Correlation increased by approximately 5–6% for RV and 5–7% for HRV, with gains observed relative to both the CNN model of Addeh, Vega, et al. (2025) and the CNN model of Salas et al. (2021). Larger relative improvements were observed for error-based and temporal alignment metrics, with MAE, MSE, and DTW reduced by approximately 7–10%, indicating lower absolute reconstruction error and improved temporal fidelity. These performance differences across architectures were statistically significant (Friedman test, p < 0.001). The bi-LSTM model achieved intermediate performance across metrics, while the proposed architecture consistently outperformed all baselines under identical preprocessing, temporal resolution, and input conditions. Although the absolute differences between architectures may appear modest, the observed improvements are consistent across correlation, error-based, and temporal alignment metrics, indicating a meaningful architectural advantage rather than a purely statistical effect.

**Table 1.** Comparison of RV and HRV reconstruction performance across different machine learning architectures on the HCP-YA dataset.

METHOD	Temporal context	Signal	MAE	MSE	r	DTW
1D-CNN + GRU (proposed)	50 s	RV	0.194 ± 0.0046	0.078 ± 0.0074	0.650 ± 0.026	53.31 ± 0.70
		HRV	0.164 ± 0.0084	0.070 ± 0.0067	0.642 ± 0.038	35.36 ± 0.92
1D-CNN Addeh, Vega, et al. (2025)	50 s	RV	0.208 ± 0.0053	0.083 ± 0.0048	0.614 ± 0.042	58.34 ± 0.84
		HRV	0.175 ± 0.0072	0.075 ± 0.0069	0.609 ± 0.065	37.22 ± 0.76
1D-CNN Salas et al. (2021)	50 s	RV	0.211 ± 0.0028	0.085 ± 0.0036	0.613 ± 0.075	59.12 ± 0.91
		HRV	0.179 ± 0.0095	0.078 ± 0.0078	0.602 ± 0.091	38.13 ± 0.81
bi-LSTM Bayrak et al. (2025)	Full sequence	RV	0.206 ± 0.0064	0.082 ± 0.0053	0.621 ± 0.064	56.07 ± 0.69
		HRV	0.173 ± 0.0093	0.074 ± 0.0047	0.615 ± 0.049	36.93 ± 0.52

All models were trained and evaluated using the native temporal resolution (TR = 0.72 s) without temporal downsampling and with matched input features. Window-based models were evaluated using a fixed temporal context of 50 s, whereas the bi-LSTM model operates in a sequence-to-sequence manner over the full fMRI time series.

Importantly, the same architectural comparison was repeated on the HCP-D and HCP-A cohorts, yielding consistent performance trends across all evaluation metrics, with the proposed 1D-CNN + GRU model consistently outperforming the baseline architectures (see Supplementary Materials, Section 9).

**Fig. 10.**
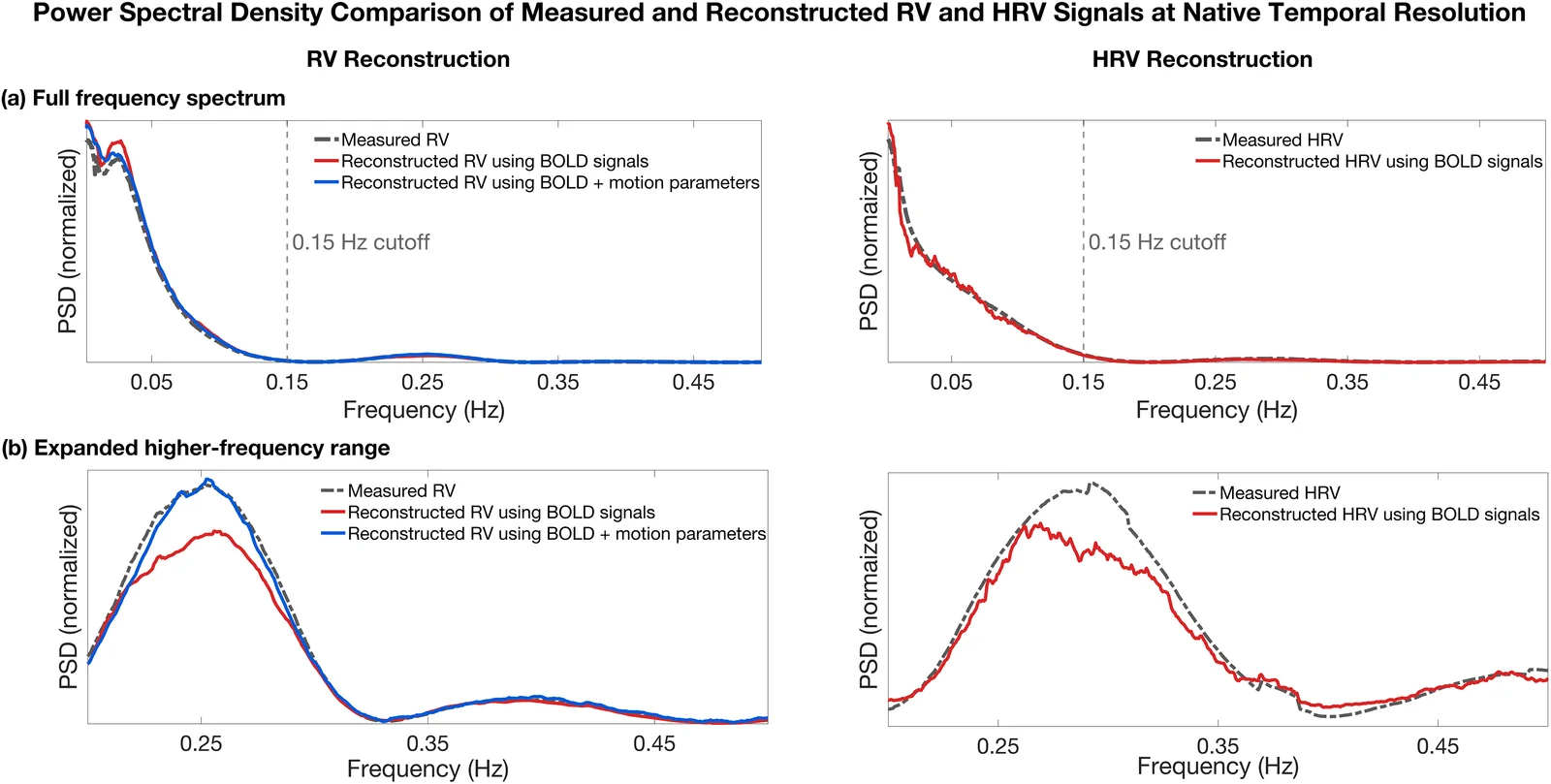
Power spectral density (PSD) comparison of measured and reconstructed RV and HRV signals at native temporal resolution. The left column shows RV reconstruction results, and the right column shows HRV reconstruction results. The top row presents the full frequency spectrum, while the bottom row presents an expanded view of the higher-frequency range. The dashed vertical line indicates the conventional 0.15 Hz cutoff commonly used in low-frequency physiological analyses. The reconstructed RV and HRV signals preserved spectral characteristics across both low- and higher-frequency ranges, including physiologically relevant fluctuations above 0.15 Hz. Inclusion of head motion parameters improved preservation of higher-frequency RV spectral content and produced closer agreement with the measured RV spectrum.

To further evaluate whether the reconstructed physiological signals preserved higher-frequency spectral content, we compared the power spectral density (PSD) of measured and reconstructed RV and HRV signals at native temporal resolution (Fig. 10). The reconstructed RV and HRV signals preserved spectral characteristics across both low- and higher-frequency ranges, including components above the conventional 0.15 Hz cutoff. For RV reconstruction, inclusion of head motion parameters improved preservation of higher-frequency spectral content and produced closer agreement with the measured RV spectrum. These findings support that the proposed framework preserves a broader physiological frequency spectrum without relying on temporal downsampling or aggressive low-pass filtering.

**Fig. 11.**
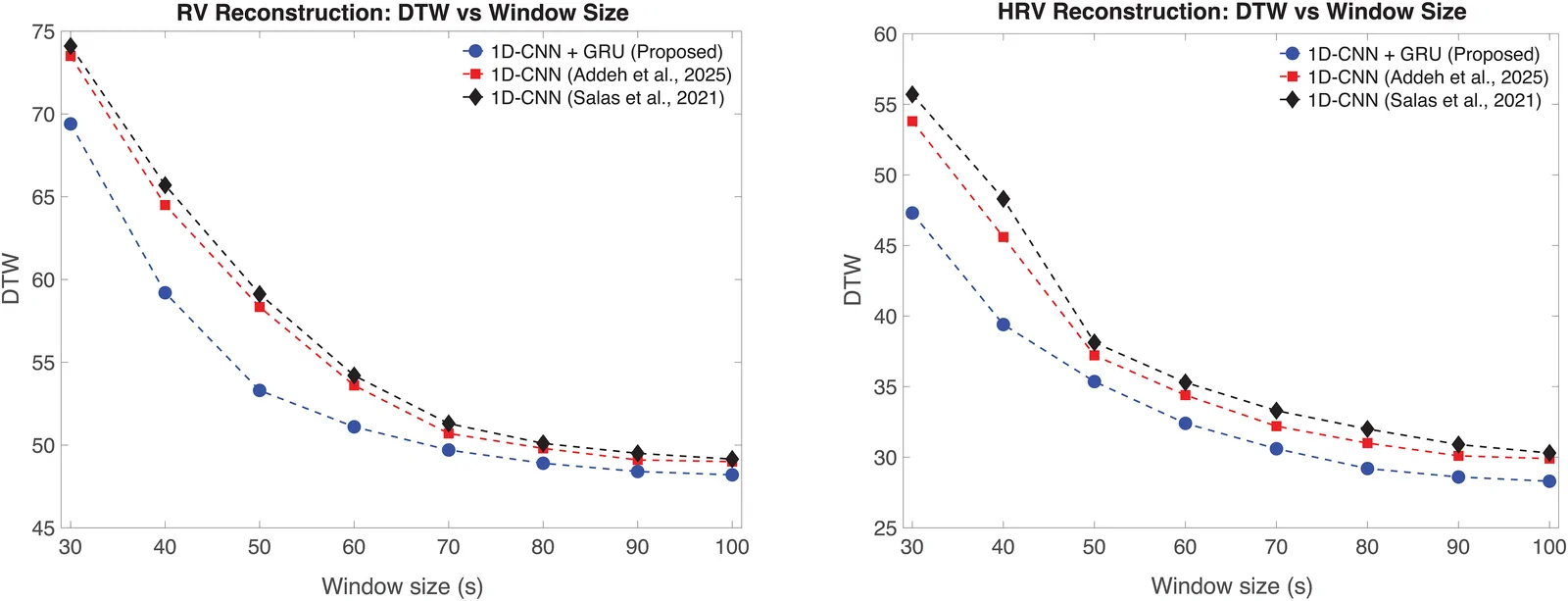
Effect of temporal window length on reconstruction performance across architectures in the HCP-YA cohort. DTW is shown as a function of window size (30–100 s) for respiratory variation (RV; left) and heart rate variability (HRV; right). CNN-only architectures exhibit a strong dependence on window length, with DTW decreasing steadily as temporal context increases. In contrast, the proposed 1D-CNN + GRU model achieves lower DTW at shorter window lengths and demonstrates earlier performance saturation, indicating reduced reliance on extended temporal context. This pattern is consistent across both physiological signals and is more pronounced for HRV, which exhibits sharper temporal dynamics.

To further examine whether the observed performance gains reflect enhanced temporal modeling rather than insufficient optimization of CNN-only baselines, we performed an additional sensitivity analysis evaluating reconstruction performance as a function of temporal window length (30–100 s) in the HCP-YA cohort, which is used here as a representative example. As shown in Figure 11, CNN-based architectures exhibited a strong dependence on window size for both RV and HRV reconstruction, with DTW decreasing steadily as temporal context increased. In contrast, the proposed 1D-CNN + GRU framework achieved lower DTW at shorter window lengths and demonstrated earlier performance saturation, indicating reduced reliance on extended temporal context. This pattern was observed consistently for both physiological signals and was particularly pronounced for HRV, which exhibits sharper temporal dynamics. Correspondingly, paired effect sizes were largest at shorter windows (dz > 1 up to 70 s), decreased at intermediate windows, and became small once performance converged at longer window lengths. Across all window sizes, performance differences between architectures were statistically significant (Friedman test, p < 0.001). Together, these findings support the interpretation that the recurrent component contributes to explicit modeling of longer-range temporal dependencies, rather than performance improvements arising solely from increased input window size in CNN-only models.

### 3.6 Evaluation of the proposed method on independent datasets

To evaluate the generalizability of the proposed method beyond the Human Connectome Project, we tested its performance on two independent datasets composed of young adult participants. Dataset 1 included 70 fMRI scans acquired during a breath-hold task (Zvolanek et al., 2023), of which 41 scans were retained after excluding cases with incomplete or corrupted respiratory recordings. Dataset 2 comprised 461 resting-state fMRI scans, with 381 retained for analysis based on the availability of usable respiratory traces (Spreng et al., 2022). Detailed inclusion criteria and preprocessing procedures are provided in Section 10 of the Supplementary Materials.

Since both datasets involve young adults, the model was trained using the HCP-YA cohort. To ensure compatibility across acquisition protocols, the HCP-YA data were resampled to match the TRs of Dataset 1 and Dataset 2. The model was then retrained on the resampled HCP-YA dataset and evaluated on the two external datasets. In the current implementation, a separate TR-matched training procedure was performed for each target temporal resolution. However, the same high-quality HCP-YA dataset was reused across datasets through controlled temporal adaptation while preserving the native temporal structure of the external datasets.

In all cases, model inputs were constructed using a fixed temporal window of 50 s, with the number of samples adjusted according to TR, ensuring consistent temporal context across datasets with different acquisition resolutions. In all cases, the input to the model included 630 mean BOLD signals; head motion parameters were included for RV reconstruction only.

**Fig. 12.**
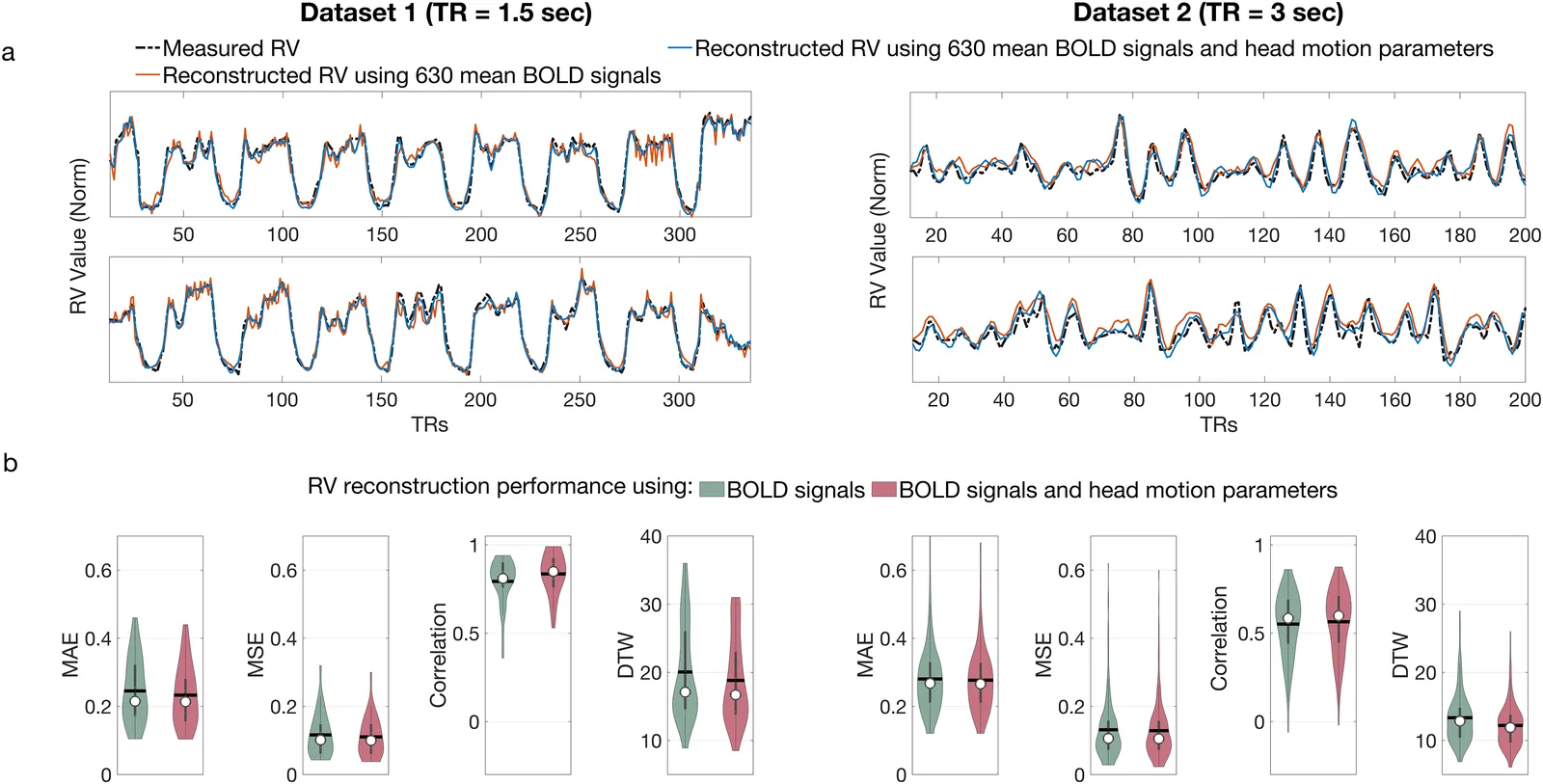
RV reconstruction performance on two independent datasets. (a) Representative RV waveforms from Dataset 1 (TR = 1.5 s) and Dataset 2 (TR = 3 s), showing measured RV (dashed black), RV reconstructed using 630 BOLD signals (orange), and using BOLD + head motion inputs (blue). (b) Violin plots summarizing performance metrics (MAE, MSE, correlation, and DTW) across subjects. Combining head motion parameters with BOLD inputs consistently improved performance.

**Fig. 13.**
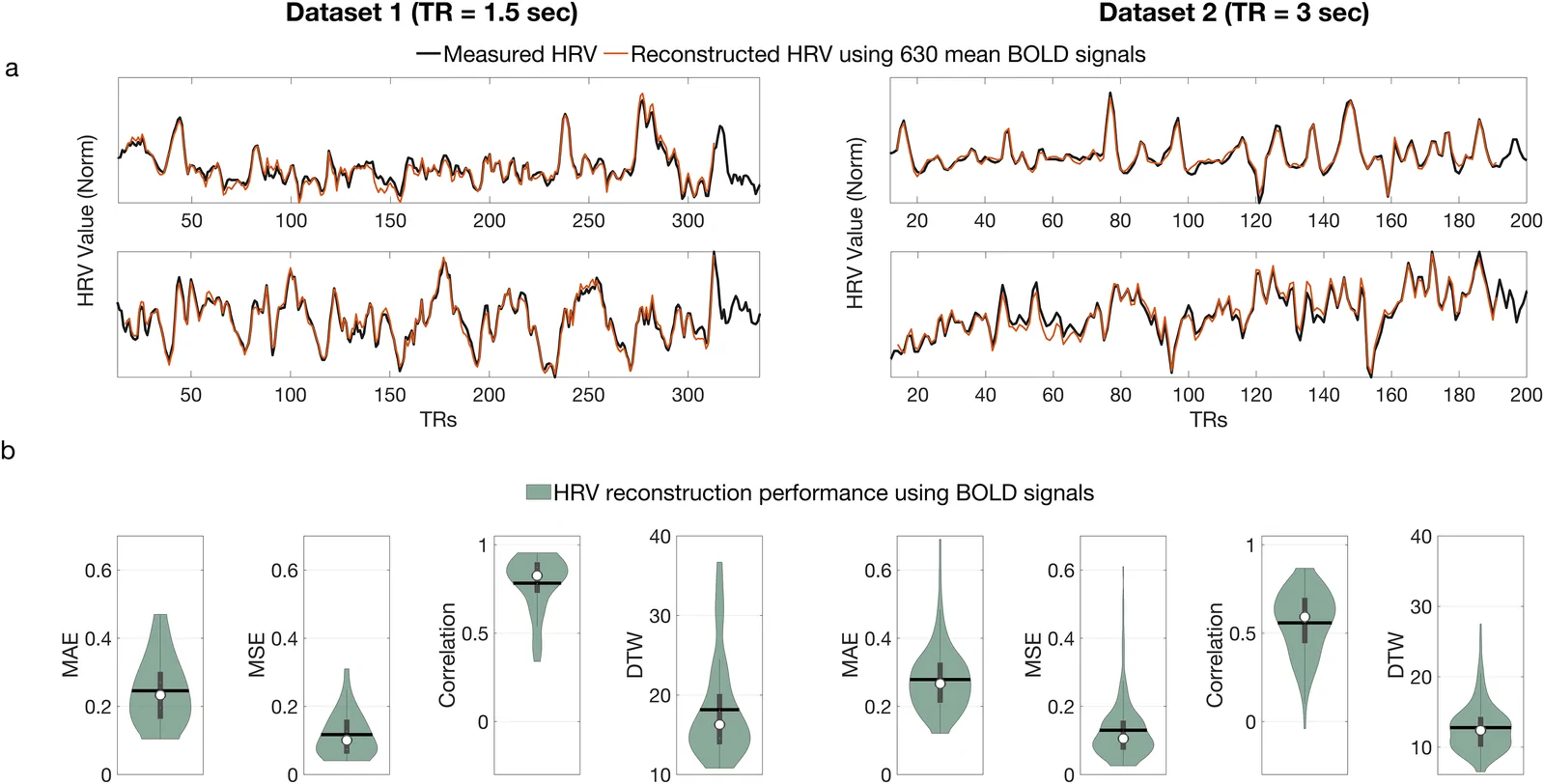
HRV reconstruction performance on two independent datasets. (a) Representative HRV waveforms from Dataset 1 (TR = 1.5 s) and Dataset 2 (TR = 3 s), comparing measured HRV (black) and HRV reconstructed using 630 BOLD signals (orange). (b) Violin plots display the distribution of reconstruction performance metrics (MAE, MSE, correlation, and DTW) across subjects. The results confirm reliable HRV estimation from resting-state BOLD signals in both datasets.

The results of the RV and HRV reconstruction are presented in Figures 12 and 13, respectively. Panels (a) display representative waveform reconstructions for both datasets, demonstrating the model’s ability to track underlying physiological dynamics in diverse scanning conditions. Panels (b) show violin plots summarizing model performance across subjects using four evaluation metrics: MAE, MSE, Pearson’s r, and DTW. As expected, reconstruction performance was lower for the dataset acquired at TR = 3 s across all evaluation metrics. As expected, correlation values were higher for Dataset 1, which was acquired during a breath-hold task, compared with Dataset 2, which consists of resting-state data with more irregular respiratory patterns and lower temporal resolution.

Overall, the results demonstrate the robustness of the proposed method across independent datasets with different acquisition parameters, task designs, and physiological characteristics, while also highlighting expected performance differences associated with temporal resolution and task structure. These findings underscore the model’s robustness and practical utility in broader neuroimaging applications, particularly under conditions of irregular breathing or non-compliance, as observed in Zvolanek et al. (2023) and Spreng et al. (2022). Quantitative reconstruction performance for both datasets, including MAE, MSE, Pearson’s r, and DTW for RV and HRV, is summarized in Table S.4 (Supplementary Materials, Section 11).

## 4 Discussion

This study introduces a hybrid deep learning framework for reconstructing RV and HRV directly from resting-state BOLD fMRI data. Our approach integrates spatially enriched inputs, drawn from dynamic functional connectivity-based and tissue-specific ROIs, with temporally informed modeling via a combined 1D-CNN + GRU architecture.

Our proposed model builds upon prior work demonstrating that physiological signals such as RV and HRV can be reconstructed from fMRI data using machine learning. Our work, in line with previous efforts, targets the frequent absence or corruption of external physiological recordings in fMRI datasets, with an emphasis on advancing reconstruction accuracy and robustness across datasets and populations. We show that meaningful RV and HRV waveforms can be accurately reconstructed using only fMRI-derived signals, achieving performance comparable with models trained on externally recorded signals. Across all evaluations—including the HCP-YA, HCP-D, and HCP-A datasets, as well as two independent external datasets—our model consistently produced high-fidelity estimates of RV and HRV. These findings support the integration of machine learning-derived physiological signals into standard preprocessing pipelines, particularly in large-scale or legacy datasets where physiological recordings are often unavailable.

### 4.1 Contribution of spatiotemporal features and ROI configuration

The improved performance observed with our combined 1D-CNN+GRU architecture highlights the importance of both spatial and temporal modeling in physiological signal reconstruction. Convolutional layers capture localized fluctuations within each time series, such as short bursts in respiration or cardiac pulses, while GRUs encode temporal dependencies over broader time windows, critical for modeling periodic or slowly varying physiological signals. Notably, the inclusion of head motion parameters enhanced RV estimation, particularly during periods of pronounced physiological variability, consistent with the known link between respiration and pseudomotion artifacts in fMRI.

The quantitative results of the ROI configuration analysis further clarify how these spatial design choices translate into measurable performance gains. As shown in Figure 5, Configuration 4 produced consistent improvements over both baseline configurations across all evaluation metrics, but with markedly different magnitudes for RV and HRV reconstruction. For RV, improvements were statistically significant yet modest in size, with mean percent differences on the order of 4–6% and small-to-moderate standardized effect sizes (Cohen’s dz). These results indicate that while the inclusion of WM and CSF regions enhances RV reconstruction, much of the respiration-related variance is already captured by cortical signals and head motion parameters. In contrast, HRV reconstruction exhibited substantially larger improvements, with percent differences ranging from approximately 6 to 21% and moderate-to-large effect sizes across all metrics. This divergence suggests that cardiac-related physiological fluctuations are more strongly dependent on both expanded spatial coverage and functionally homogeneous parcellation strategies. Together, these findings demonstrate that the benefits of atlas selection are signal specific: dynamic functional parcellations combined with WM and CSF regions yield incremental gains for RV but are critical for robust HRV reconstruction.

Beyond model architecture, the choice of spatial atlas played a central role in reconstruction performance. Configuration 4, which combines dFC-based cortical parcellations with white matter and CSF ROIs, consistently outperformed other configurations. Importantly, while the larger number of ROIs in this configuration may contribute to improved performance by increasing spatial sampling, the observed gains cannot be explained by ROI count alone. Configuration 2, which employs the same dFC-based cortical atlas without white matter and CSF regions, and Configuration 3, which includes white matter and CSF but relies on a conventional multi-modal cortical atlas, both showed lower performance. These comparisons indicate that neither increased ROI count nor inclusion of non-gray matter regions alone is sufficient. Instead, optimal performance arises from the combination of functionally homogeneous dFC-based cortical ROIs with white matter and CSF regions that carry strong physiological modulation.

The white matter and CSF contain stronger respiratory and cardiac influences in resting-state fMRI because of their vascular architecture, partial-volume effects, and proximity to major fluid compartments. As a result, they provide physiologically informative signals that are not captured by cortical ROIs alone. Incorporating white matter and CSF ROIs also raises the question of whether genuine neural activity in white matter could confound physiological modeling. Although white matter BOLD signals can include neural components such as slow axonal conduction-related activity or glial metabolic fluctuations (Grajauskas et al., 2019), these neural signals are typically weaker, less periodic, and less temporally aligned with respiratory and cardiac rhythms than the fluctuations driven by physiological processes. Our ROI-based averaging strategy further reduces the influence of localized or spatially heterogeneous neural activity because averaging across large regions attenuates small, focal neural fluctuations while preserving global physiological variations that manifest coherently across broad spatial scales. The supervised learning objective then emphasizes only the components that consistently covary with the measured RV and HRV signals, while neural fluctuations that do not follow physiological timing or frequency structure contribute minimally to the learned mapping. The inclusion of CSF ROIs, which contain negligible neural activity and strong physiological pulsatility, provides an additional physiologically dominated reference that stabilizes the representation and reduces any residual neural influence in white matter. Together, these factors explain why combining gray matter, white matter, and CSF ROIs consistently enhances RV and HRV reconstruction across cohorts and support the interpretation that the most informative variance in these regions arises from physiological rather than neural sources. Explicit separation of physiological and neural contributions, through multimodal recordings or interpretability analyses, remains an important direction for future research.

### 4.2 Age-related physiological and neurovascular factors affecting RV and HRV reconstruction

Reconstruction of RV and HRV from BOLD fMRI is influenced by age-related differences in physiological dynamics and neurovascular coupling, including changes in vascular compliance, autonomic regulation, and the temporal characteristics of physiological fluctuations across the lifespan. Pediatric data are typically characterized by higher physiological variability and increased motion, whereas aging populations exhibit slower and smoother physiological rhythms, often accompanied by altered cerebrovascular responsiveness. Although the resulting performance gains from age-specific architectural tuning are modest (approximately 1–2% across MAE, MSE, Pearson’s r, and DTW), such differences are meaningful in the context of physiological confound modeling, where small residual errors can propagate into downstream denoising and functional connectivity analyses. The consistent advantage of cohort-optimized architectures observed across all age groups, therefore, reflects improved alignment between model design and age-dependent physiological and BOLD signal properties, rather than overfitting or dataset-specific effects. Future work will further investigate age-aware modeling strategies that explicitly incorporate neurovascular and autonomic variability to achieve additional gains in physiological reconstruction accuracy across the lifespan.

### 4.3 Impact of model architecture and temporal resolution

The comparative analysis of model architectures highlights the importance of aligning network design with both the temporal characteristics of physiological signals and the native sampling properties of fMRI data. When evaluated under identical preprocessing conditions using the native temporal resolution, the proposed 1D-CNN + GRU framework consistently outperformed CNN-only baselines and achieved higher reconstruction accuracy than a sequence-to-sequence bi-LSTM model, despite operating on a substantially shorter temporal context.

A key distinction between the proposed approach and several prior methods lies in the treatment of temporal resolution and frequency content. Earlier studies, including the CNN model of Salas et al. (2021) and the bi-LSTM framework of Bayrak et al. (2025), relied on temporally downsampled fMRI data (effective TR = 1.44 s) and applied band-limited filtering, typically restricting the reconstructed signals to the 0.01–0.15 Hz range. While such preprocessing reduces high-frequency noise and can increase apparent signal-to-noise ratio, it also suppresses physiologically meaningful temporal variability. Analysis of the native-TR data in the present study demonstrates that both RV and HRV contain non-negligible spectral power above 0.15 Hz (see Supplementary Materials, Section 8), reflecting faster modulations and transient dynamics that are attenuated or removed by downsampling and low-pass filtering.

By operating directly on the native temporal resolution without frequency-restricted filtering, the proposed model preserves the full temporal bandwidth of the BOLD signal, enabling more faithful alignment between fMRI-derived features and underlying physiological processes. This setting represents a more challenging reconstruction task, as higher-frequency components increase variability and reduce temporal smoothness, but it more accurately reflects modern multiband fMRI acquisitions, where sub-second repetition times improve sampling of physiological fluctuations and respiration-related pseudomotion effects. In addition, the framework was evaluated across datasets with heterogeneous acquisition characteristics, including differences in repetition time, task structure (resting-state versus breath-hold), physiological variability, motion characteristics, and signal quality. While many earlier fMRI studies were acquired with relatively long repetition times, recent advances in multiband EPI have led to a growing number of large-scale datasets being collected at high temporal resolution, increasing the importance of reconstruction methods that operate directly on native-resolution fMRI data.

Architecturally, the results further indicate that hybrid models that decouple local feature extraction from longer-range temporal modeling are well suited for physiological signal reconstruction under constrained temporal windows. CNN-only models rely on repeated pooling operations to progressively compress temporal information, which can limit sensitivity to delayed or nonstationary physiological effects when short windows are used. In contrast, the proposed architecture leverages convolutional layers to extract spatially and temporally localized features while employing a GRU to explicitly model temporal dependencies across the full 50-s window. This design enables the capture of known physiological delays, such as the 10–20 s lag between respiratory changes and their hemodynamic effects, without requiring access to the entire fMRI time series.

The pattern of performance gains across evaluation metrics further supports this interpretation. While improvements in correlation were moderate, larger relative gains were consistently observed for error-based and temporal alignment metrics (MAE, MSE, and DTW). These metrics are more sensitive to absolute amplitude deviations and local temporal misalignment, which are directly affected by how effectively temporal dependencies are modeled. The inclusion of the GRU component, therefore, appears particularly beneficial for reducing transient prediction errors and improving temporal fidelity, even when overall waveform similarity, as captured by correlation, is already high.

The bi-LSTM model, which performs sequence-to-sequence reconstruction over complete scans, achieved competitive but lower performance under native-TR conditions. Although full-sequence models benefit from extended temporal context, they also introduce asymmetries at the beginning and end of the time series and may be more sensitive to inter-scan variability and nonstationarities. In addition, sequence-to-sequence architectures incur higher computational cost and reduced flexibility when applied to scans of varying duration.

In this context, using a fixed 50-s temporal window provides a physiologically meaningful and interpretable framework for RV and HRV reconstruction, capturing delayed hemodynamic effects without introducing unnecessary long-range dependencies. Compared with full-sequence models, window-based approaches offer improved temporal interpretability, reduced sensitivity to scan length, and greater practical flexibility, while maintaining competitive reconstruction accuracy.

Together, these findings indicate that reconstruction accuracy is not determined solely by model complexity or temporal coverage, but by how effectively the architecture exploits physiologically meaningful information at the native temporal resolution of the data. Accordingly, direct numerical comparisons with results obtained under different temporal resolutions and preprocessing strategies should be interpreted with caution, as such differences can substantially influence reported performance metrics. The proposed 1D-CNN + GRU framework achieves a favorable balance between temporal context, architectural expressiveness, and computational efficiency, making it particularly well suited for retrospective physiological reconstruction in fMRI datasets where preserving native temporal dynamics is critical.

### 4.4 Role of head motion in physiological signal reconstruction

In this work, head motion parameters were intentionally not regressed from the BOLD time series prior to RV and HRV reconstruction, but instead provided as auxiliary inputs to the learning framework. This design choice builds directly on our previous work, where we demonstrated that apparent head motion in fMRI contains both true rigid-body motion and respiration-related pseudomotion, and that a joint representation of BOLD signals and head motion parameters enables machine learning models to distinguish between these components rather than conflating them (Addeh, Vega, et al., 2025).

Respiration-induced chest wall motion and associated magnetic field perturbations can produce systematic, respiration-synchronous fluctuations in estimated head motion parameters, which carry physiologically meaningful information relevant to RV estimation. Removing motion-related variance a priori would, therefore, risk discarding informative BOLD components associated with physiological fluctuations. By retaining both BOLD signals and motion parameters as inputs, the model is allowed to learn whether and how motion-related signals contribute to physiological reconstruction, rather than imposing a hard separation through preprocessing. This perspective aligns with recent studies demonstrating reconstruction of physiological signals from fMRI without explicit regression of motion-related variance (Addeh et al., 2023; Addeh, Vega, et al., 2025; Bayrak et al., 2021, 2025; Salas et al., 2021).

Importantly, while our prior work established that head motion parameters contain respiration-related physiological information that can aid RV estimation when used as auxiliary inputs (Addeh, Vega, et al., 2025), the present findings extend this insight within a substantially richer modeling context. Here, motion parameters are evaluated in conjunction with a spatially enriched ROI representation spanning cortical, subcortical, white matter, and CSF regions, and within a unified 1D-CNN + GRU architecture designed to capture both short- and long-range temporal dependencies. The observed improvements indicate that motion parameters contribute complementary physiological information even when strong respiration-related cues are already present in the BOLD signal, rather than merely recapitulating earlier results obtained under more limited spatial or architectural settings.

In parallel, downstream functional connectivity analyses applied standard nuisance regression, including head motion parameters, to ensure that motion-related variance did not confound connectivity estimates. This separation between physiological signal reconstruction and connectivity analysis allows motion-related effects to be retained where informative for RV estimation, while still adhering to established practices for motion control in functional connectivity analyses.

### 4.5 Age-specific model performance across the lifespan

By adapting the model architecture and hyperparameters for each HCP cohort (developmental, young adult, and aging), we demonstrate the applicability of the proposed framework across the human lifespan. Despite substantial developmental, neurovascular, and physiological variability, the model achieved robust reconstruction performance within each age group. In pediatric populations, physiological confounds may be more complex due to higher motion levels and rapid developmental changes, while in older adults, vascular stiffness and altered autoregulatory mechanisms may modulate the BOLD–physiology relationship. Our results suggest that these population-specific characteristics can be effectively captured with appropriate model tuning.

### 4.6 Transferability to independent datasets

The proposed model demonstrated robust cross-dataset performance on two independent datasets acquired using different scanning protocols and repetition times. These results indicate that the learned relationship between BOLD-derived features and physiological waveforms is not specific to a single dataset or acquisition scheme, but instead captures underlying physiological dynamics that generalize across studies.

Differences in repetition time across datasets were addressed by temporally adapting the training data to match the acquisition TRs of the external datasets, rather than modifying the external data themselves. This design choice avoids upsampling lower temporal resolution data and does not imply that a unique, dataset-specific model must be trained for each study. Instead, it provides a practical mechanism for reusing a single high-quality training dataset across heterogeneous acquisition protocols while preserving physiological signal fidelity.

In the current implementation, this strategy required retraining the model after adapting the HCP-YA training data to each target TR. However, the framework should be viewed as TR-adaptive rather than dataset-specific, since the same source training dataset was reused across acquisition protocols without modifying the external datasets themselves. Importantly, preserving the native temporal structure of the external datasets avoids artificially increasing the sampling rate of lower temporal resolution data through interpolation. Because interpolation does not increase the true temporal resolution of the physiological signal, evaluation was performed using the native acquisition characteristics of each external dataset. Future work may explore multi-TR or TR-invariant training strategies to enable a single model to generalize across multiple temporal resolutions without retraining. Although overall performance remained robust across datasets, a modest reduction in reconstruction accuracy was observed for data acquired at longer repetition times (e.g., TR = 3 s). This effect is not attributable to differences in temporal window length, as a fixed window duration of 50 s was used across all datasets with the number of samples adjusted according to TR. Instead, the reduced performance primarily reflects the lower temporal sampling rate, which results in fewer samples within the fixed temporal window and limits the model’s ability to resolve rapid physiological fluctuations, particularly for HRV. While temporal downsampling or lower sampling rates may sometimes increase correlation metrics by smoothing high-frequency variability and reducing signal complexity, they also remove physiologically meaningful temporal information that is important for accurate waveform reconstruction. Accordingly, preservation of native temporal resolution preserves richer physiological content despite increasing reconstruction difficulty. This behavior is consistent with the known sensitivity of physiological signal reconstruction to temporal resolution and does not detract from the model’s ability to generalize across heterogeneous acquisition protocols.

In addition, higher correlation values and lower reconstruction errors observed for Dataset 1 are likely attributable to the breath-hold task, which induces large, structured respiratory fluctuations that are easier to reconstruct from BOLD signals. In contrast, resting-state datasets exhibit more irregular breathing patterns and weaker physiological modulation, leading to reduced correlation despite comparable overall reconstruction fidelity.

### 4.7 Functional connectivity implications of reconstructed physiology

While RV and HRV reconstruction achieved moderate-to-strong agreement at the time-series level (mean Pearson r~0.65), the downstream impact on resting-state FC was substantially stronger. Across the HCP-D, HCP-YA, and HCP-A cohorts, FC matrices corrected using reconstructed RV/HRV exhibited high similarity to those obtained using measured physiological recordings, with mean FC similarity values ranging from approximately 0.83 to 0.86 and corresponding ∆FC similarity values around 0.80–0.81. These results demonstrate that reconstructed physiological regressors yield FC estimates that closely match those derived from recorded physiology.

This apparent dissociation between waveform-level reconstruction accuracy and FC-level agreement reflects a fundamental distinction between pointwise signal fidelity and the requirements of physiological confound correction. FC is primarily sensitive to the shared variance structure introduced by physiological fluctuations across regions, rather than to exact temporal correspondence of individual physiological samples. Consequently, reconstructed RV/HRV signals do not need to perfectly reproduce the original waveforms to effectively capture and remove connectivity-relevant physiological variance.

Importantly, the strong agreement observed at the FC level likely reflects the ability of the proposed framework to accurately capture prominent physiological events, including large respiratory excursions (deep breaths) and pronounced heart rate fluctuations (e.g., transient accelerations or decelerations), both of which are known to exert a disproportionate influence on resting-state connectivity. Although reconstruction accuracy averaged across the entire time series was moderate, high-amplitude RV and HRV fluctuations that are most strongly coupled to widespread BOLD and global signal effects were reliably recovered. As these events dominate the connectivity-relevant physiological variance, their accurate reconstruction is sufficient to yield FC corrections that closely resemble those obtained using measured physiology.

This interpretation is further supported by the ∆FC analyses, which directly assess correction-induced changes relative to baseline FC. High ∆FC similarity across cohorts indicates that reconstructed RV/HRV removes essentially the same connectivity component as measured physiological regressors. The small magnitude and spatially diffuse nature of the difference maps between estimated- and measured-corrected FC further suggest an absence of systematic bias introduced by the reconstruction process.

Notably, FC and ∆FC similarity were highest in the young adult cohort, with slightly reduced agreement observed in pediatric and aging cohorts. This pattern is consistent with increased motion, and cohort-specific hemodynamic differences in children and older adults, which may introduce additional variability in physiological correction effects. Nevertheless, reconstructed RV/HRV consistently captured the dominant physiological contributions to FC across all cohorts.

Together, these findings align with prior work demonstrating that predicted physiological regressors can yield FC corrections comparable with those obtained using external recordings, despite imperfect waveform-level agreement (e.g., Bayrak et al., 2025). From a practical perspective, this distinction is critical: many large-scale or legacy fMRI datasets lack reliable physiological recordings, limiting the applicability of conventional physiology-based correction methods. The present results demonstrate that reconstructed RV/HRV can serve as effective substitutes for measured signals in FC analyses, enabling retrospective physiological correction while preserving the integrity of functional connectivity estimates.

### 4.8 Limitations and future directions

While the proposed method offers substantial benefits, several limitations warrant consideration. First, unlike RV reconstruction, head motion parameters did not improve HRV estimation. This likely reflects the weaker coupling between cardiac pulsatility and gross head movements during resting-state scans. Future work could explore alternative physiological proxies, such as cerebrospinal fluid inflow dynamics or venous pulsatility, which may provide more direct sensitivity to cardiac-related fluctuations.

A second limitation concerns partial HRV reconstruction due to temporal context requirements. Whereas RV could be reconstructed using past-only, future-only, or bidirectional input windows, HRV estimation was effective only when both past and future BOLD signals were available. Attempts to reconstruct HRV using unidirectional windows resulted in reduced accuracy (see Supplementary Materials, Section 12), likely due to the sharper and more transient nature of cardiac-induced BOLD fluctuations. In contrast, RV exhibits slower dynamics and explains a larger proportion of BOLD variance across widespread brain regions, making it more amenable to reconstruction from limited temporal context (Chang & Glover, 2009; Golestani et al., 2015). As a result, HRV reconstruction is not feasible at the beginning and end of each scan, where full temporal context is unavailable, leading to edge effects and incomplete waveform coverage. Future studies could investigate adaptive windowing strategies or causal modeling approaches to mitigate this limitation.

An additional limitation of the present study is the absence of neonatal fMRI data. While the Developing Human Connectome Project (dHCP) provides high-quality neonatal imaging alongside physiological measurements, meaningful inclusion of this cohort would require specialized preprocessing pipelines, stringent motion and physiological quality control, and age-specific model adaptations to account for the unique neurovascular and anatomical properties of the neonatal brain. As such, incorporation of dHCP data was beyond the scope of the current study. Extending the proposed framework to neonatal populations represents an important and natural direction for future work and would further strengthen its lifespan applicability.

Model interpretability represents an important consideration for machine learning-based physiological reconstruction. While the proposed framework is not explicitly designed as an interpretable model, we address interpretability through a series of input- and architecture-level analyses. Specifically, ROI configuration comparisons and network-level ablation experiments provide insight into which tissue classes and functional networks contribute most strongly to reconstruction performance, while temporal window sensitivity analyses clarify the temporal scales most relevant for modeling RV and HRV. Together, these analyses offer a practical and physiologically grounded interpretation of the model’s behavior without relying on post-hoc gradient-based explainability methods, which can be unstable in highly correlated fMRI inputs. More fine-grained filter-level or saliency-based analyses remain an important direction for future work.

In addition, although the sliding-window strategy implicitly captures hemodynamic delays through temporally extended inputs, explicit modeling of the physiological-to-BOLD transfer function may further improve reconstruction accuracy. Integrating physiologically informed delay models or learned response functions represents a promising direction for future work.

Finally, while the proposed framework is computationally efficient for offline analysis, real-time deployment would require additional engineering considerations. Applications such as neurofeedback or intraoperative monitoring would necessitate causal implementations and low-latency processing pipelines, which remain topics for future investigation.

## 5 Conclusion

This study introduces and validates a flexible approach that demonstrates robust performance across age groups and acquisition protocols for reconstructing key physiological fluctuations from resting-state fMRI data using deep learning. By leveraging rich spatial and temporal features, the proposed model enables accurate recovery of RV and HRV across diverse age groups and acquisition protocols. These reconstructed signals can be directly integrated into fMRI preprocessing pipelines for physiological confound correction, offering a powerful alternative to external recordings and enhancing the utility of large-scale neuroimaging datasets.

## Supplementary Material

Supplementary Material

## Data Availability

The datasets analyzed in this study are publicly available through the Human Connectome Project (HCP), including the Human Connectome Project-Development (HCP-D), Human Connectome Project-Young Adult (HCP-YA), and Human Connectome Project-Aging (HCP-A) datasets. The code implementing the proposed method is available on GitHub at https://github.com/jaliladde/fMRI-Physio-Reconstruction
