# Supplementary material for: Improving respiratory and heart rate variation estimation from resting-state BOLD fMRI across the lifespan using a functionally informed, tissue-aware deep learning framework
Source: Imaging Neurosci (Camb). 2026 Aug 27;4:IMAG.a.1346. doi: 10.1162/IMAG.a.1346 (PMC13522955; doi:10.1162/IMAG.a.1346)
Supplement: Supplementary file 1 [file IMAG.a.1346_supp.pdf]

Supplementary Materials

1. Datasets and Preprocessing

1.1. Datasets

1.1.1. HCP

To evaluate our method across the lifespan, we used three major datasets from the Human Connectome Project (HCP): HCP in Development (HCP-D), HCP in Young Adults (HCP-YA), and HCP in Aging (HCP-A). All datasets include high-quality resting-state fMRI scans acquired using Siemens 3T Prisma scanners with multiband acceleration, 2 mm isotropic voxels, and TRs of 720-800 ms.

The HCP-YA dataset includes healthy adults aged 22–35 years. Each subject underwent four 14.4-minute resting-state scans (1200 volumes per run) with left→right (LR) and right→left (RL) phase encoding. The fMRI data were acquired using a 2D multiband GRE-EPI sequence (TR = 720 ms), and physiological recordings included respiratory signals captured via an abdominal belt and cardiac signals via photoplethysmography (PPG), both sampled at 400 Hz (Van Essen et al., 2013).

The HCP-D dataset extends coverage to children and adolescents aged 5–21 years. Due to increased motion and compliance challenges in this population, sessions included two 6.5-minute scans per visit with anterior→posterior (AP) and posterior→anterior (PA) phase encoding (TR = 800 ms, TE = 37 ms). Respiratory and cardiac signals were recorded using the same belt and PPG setup as in HCP-YA, though signal quality was more variable (Harms et al., 2018).

The HCP-A dataset includes adults aged 36 to over 100 years. Acquisition parameters were harmonized with those used in HCP-D, and two resting-state runs were acquired per session with AP and PA phase encoding. As with the other cohorts, physiological signals were recorded using an abdominal respiratory belt and PPG finger sensor at 400 Hz (Harms et al., 2018).

1.1.2. Additional Datasets for Model Validation

To rigorously assess the robustness and generalizability of our approach for reconstructing RV waveforms, we expanded our analysis to include two additional datasets beyond the HCP-YA. These datasets were chosen for their distinct characteristics, particularly in terms of TR and task-induced physiological variations.

Dataset 1 comprises 70 individual scans from a targeted breath-holding task-based fMRI study, characterized by a TR of 1.5 seconds and includes a total of 340 volumes, allowing detailed observation of task-induced physiological responses (Zvolanek et al., 2023). Dataset 2 features 461 scans from a standard resting-state fMRI study with a TR of 3 seconds, covering 204 volumes per scan, providing a comprehensive view of resting-state physiological fluctuations under longer TR conditions (Spreng et al., 2022). These additional tests ensure that our model, trained on the HCP data, is capable of generalizing across different experimental setups and physiological conditions.

1.2. Quality Control of Physiological Traces

1.2.1. Respiratory Traces

In the pursuit of robust physiological monitoring, the respiration and PPG traces were found to encounter a diverse range of artifacts compromising data integrity. These interferences are multifaceted, arising from technical glitches, sensor displacement, and patient-driven adjustments, such as movement or physiological reactions like coughing and swallowing. Proper attachment of the belt to the transducer, the fitting around the subject's abdomen, and the fidelity of the transducer-subject interface are all critical to data accuracy. The phenomenon of signal clipping, resulting from the saturation of the analog-to-digital converter during intense respiratory events, further complicates the capture of the true respiratory patterns.Participant cooperation is another crucial factor in data collection. Shifts in the respiration belt's position can lead to false readings, while discomfort or induced anxiety can affect the participant's breathing. Additionally, the belt itself may restrict natural respiratory movements, contributing to the complexity of error identification.

Our study classifies the respiratory signal anomalies within the HCP dataset into seven distinct classes:

- Class 1: Respiratory signals with correctable spikes,
- Class 2: Those with non-correctable spikes,
- Class 3: Inhalations and exhalations that peak at maximum values,
- Class 4: Pronounced signal clipping,
- Class 5: Periods without recording,
- Class 6: Shifts in belt position leading to halted recording,
- Class 7: Instances of non-recording.

This classification sets the stage for more nuanced data analysis and a systematic approach to the quality assurance of respiratory signals.

1.2.2. PPG Traces

Similar to respiratory signals, PPG traces in the HCP datasets exhibit various artifacts that can compromise their usability for downstream analysis. These issues often stem from motion-induced noise, poor sensor placement, loss of contact with the skin, or hardware-related acquisition errors. Subject-related factors, such as hand movement, peripheral vasoconstriction, or restlessness, can further degrade signal quality. As with the respiratory belt, proper placement and secure attachment of the PPG sensor, typically worn on the fingertip, are essential for stable signal acquisition.

To systematically assess the quality of PPG data, we developed a six-class categorization scheme based on visual inspection and signal characteristics:

- Class 1: Signals with correctable spikes
- Class 2: Signals with uncorrectable spikes due to extreme artifacts or noise
- Class 3: Signal clipping or saturation
- Class 4: Partial recordings with intermittent gaps, likely due to temporary sensor disconnection
- Class 5: Abnormally low-amplitude traces, inconsistent with typical ppg morphology
- Class 6: Sessions with no recorded signal

This classification sets the stage for more nuanced data analysis and a systematic approach to the quality assurance of respiratory signals.

1.2.3. Signal Inclusion Criteria

Using physiological data of inadequate quality can degrade model performance and reliability. Therefore, only signals classified in the highest quality category, those with correctable spikes, were retained for further analysis. To ensure physiological completeness and consistency, a scan was included in the study only if both the respiratory and PPG traces met this quality criterion. This conservative selection strategy ensured that the input data provided sufficient temporal and morphological fidelity for accurate RV and HRV estimation.

1.2.4. Preprocessing of Physiological Recordings

Respiratory belt and PPG signals provided with the HCP datasets were sampled at 400 Hz and analyzed without additional temporal band-pass filtering. Signals were used at their native sampling rate to preserve the full physiological bandwidth and avoid introducing assumptions about frequency content beyond those inherent to the recording hardware. Spike artifact correction was performed in MATLAB using a moving-window outlier detection approach. For each physiological trace, transient spikes were identified using the

filloutliers function with a moving mean criterion and replaced using the preceding valid sample: filloutliers(signal,'previous','movmean',window, where the window length was selected based on signal type and sampling rate. This procedure was applied only to isolated, short-duration spikes and did not modify sustained artifacts or clipped segments.

Signal rejection criteria were applied following spike correction. Physiological recordings were excluded if they exhibited non-removable spikes, prolonged clipping or saturation, extended flat segments indicative of signal loss, partial recordings with substantial gaps, or complete absence of signal. Only traces classified as Class 1 (signals with correctable spikes) were retained for analysis.

After quality-based exclusion and spike correction, RV and HRV were computed only from retained high-quality traces. Prior to model training, RV and HRV targets were normalized within each scan to prevent extreme values from disproportionately influencing the loss function. No additional outlier clipping was applied beyond the quality-control procedures described above.

1.2.5. Temporal Alignment of Physiological Signals and fMRI

Derivation of physiological targets followed established definitions. RV was computed from the respiratory belt signal as the standard deviation of the waveform within a 6-s sliding window centered at each time point. HRV was derived from the PPG signal as the inverse of the mean inter-beat interval (IBI) within a 6-s sliding window centered at each time point (Chang et al., 2009). These RV and HRV time series served as ground-truth targets for supervised model training. No additional temporal filtering was applied to the respiratory belt or PPG recordings, nor to the derived RV and HRV signals.

Physiological recordings were acquired at 400 Hz, whereas fMRI data were sampled at the repetition time (TR). Temporal alignment between physiological signals and fMRI was achieved inherently through the window-based computation: for each fMRI volume, RV and HRV were calculated using a physiological signal window centered on the corresponding fMRI acquisition time. As a result, RV and HRV time series were defined directly on the fMRI temporal grid. No explicit downsampling, resampling, or temporal interpolation was applied to align physiological measurements with fMRI data.

1.3. fMRI Data Preprocessing

The fMRI images were corrected for geometric distortions caused by B0 inhomogeneity using the TOPUP tool in FSL (Andersson et al., 2003); corrected for head motion by the MCFLIRT tool in FSL (Jenkinson et al., 2002); and registered to 2 mm MNI space using Advanced Normalization Tools (ANTs) (Avants et al., 2011).

For the head motion correction, we utilized the single-band reference (SBRef) image that is captured at the onset of each scan. The SBRef image is advantageous due to its lack of slice acceleration effects and absence of T1 saturation, which typically complicate EPI images (Smith et al., 2013). This results in an image with enhanced tissue contrast and signal-to-noise ratio, making it the preferred choice for a reference in motion correction. MCFLIRT aligns each volume in the time series to the reference SBRef image through an affine transformation. This process calculates six motion parameters: three translations along the x, y, and z axes, and three rotations about the same axes, commonly referred to as pitch, yaw, and roll. These parameters are crucial for the subsequent steps in the data processing pipeline, as they provide a quantitative measure of the subject's head motion during the scan. Only scans with head motion below 1 mm in all directions were retained, provided that the corresponding physiological recordings (respiratory and PPG) also met the quality thresholds described in Section 1.2.

2. Age-Specific Model Architecture and Hyperparameter Selection

Given the age-related variability in physiological dynamics, BOLD signal properties, and head motion patterns, we designed tailored 1D-CNN + GRU models for each age group: children (HCP-D), young adults (HCP-YA), and older adults (HCP-A). These configurations, summarized in Table S.1, were designed to accommodate age-specific differences in physiological dynamics, signal complexity, and motion characteristics across the lifespan. Our goal was to optimize model capacity and regularization to best match the physiological and imaging features typical of each cohort, ensuring robust and accurate reconstruction of respiratory and cardiac indices across the lifespan.

Prior literature informed the physiological motivation underlying the cohort-specific architectural adaptations rather than prescribing exact numerical hyperparameter values. Studies of developmental and lifespan fMRI have demonstrated substantial age-related differences in motion behavior, physiological variability, and temporal structure of BOLD fluctuations (Dosenbach et al., 2010; Fair et al., 2020; Kaplan et al., 2022). These observations motivated the use of smaller kernels and larger recurrent capacity for pediatric cohorts with greater temporal variability and motion, intermediate configurations for young adults, and larger kernels with reduced recurrent complexity for aging cohorts exhibiting smoother physiological dynamics. Final hyperparameter values were selected using fixed

cohort-specific configurations chosen to provide stable training and physiologically appropriate model capacity for each age group.

2.1. Cohort-Specific Model Configurations

2.1.1. Children (HCP-D, Ages 5–21):

To accommodate the higher physiological variability and motion in pediatric data, we employed compact convolutional kernels (kernel size = 3) to capture localized, high-frequency fluctuations. A higher-capacity GRU layer (96 units) was used to model the more complex and irregular temporal dynamics. Dropout was increased to 0.4 to address the elevated inter-subject variability, and batch normalization was included after each convolutional layer to stabilize training.

2.1.2. Young Adults (HCP-YA, Ages 22–35):

In this cohort, stable physiological rhythms and low motion artifacts allowed for a more balanced architecture. Moderate kernel sizes (5), a GRU with 64 units, and a dropout rate of 0.3 achieved an optimal trade-off between learning capacity and generalization. Batch normalization continued to support convergence and mitigate internal covariate shifts.

2.1.3. Older Adults (HCP-A, Ages 36–100):

Given the smoother and slower physiological variations in aging individuals, often associated with vascular stiffening and reduced autonomic flexibility, we used larger kernels (size = 7) to extract long-range features while reducing filter complexity (32-128 filters). The GRU size was scaled down to 48 units, and the dropout rate was set to 0.3. As in other models, batch normalization helped mitigate training instability.

Table S.1. Model architecture and hyperparameter settings for RV and HRV estimation across age-specific cohorts.

Parameter	HCP-D (Children)	HCP-YA (Young Adults)	HCP-A (Aging)
Input Shape (RV)	(50/TR, 636)	(50/TR, 636)	(50/TR, 636)
Input Shape (HRV)	(50/TR, 630)	(50/TR, 630)	(50/TR, 630)
CNN Layer 1	64 filters, k=3	64 filters, k=5	32 filters, k=7
CNN Layer 2	128 filters, k=3	128 filters, k=5	64 filters, k=7
CNN Layer 3	256 filters, k=3	256 filters, k=5	128 filters, k=7
Batch Normalization	Yes	Yes	Yes
Dropout (Each Layer)	0.4	0.3	0.3
GRU Layer	96 units	64 units	48 units
GRU Return Sequences	False	False	False
Dense Hidden Layer	64 units (Leaky ReLU)	64 units (Leaky ReLU)	64 units (Leaky ReLU)
Dense Output Layer	1 unit (linear)	1 unit (linear)	1 unit (linear)
Optimizer	Adam (lr=0.001)	Adam (lr=0.001)	Adam (lr=0.001)
Loss Function	MSE	MSE	MSE
Window Size (TRs)	65	65	65
Batch Size (Suggested)	16	32	16

Figure S.1 provides a schematic illustration of the proposed 1D-CNN + GRU architecture for RV reconstruction in the HCP-YA cohort, serving as a representative example of the overall modeling framework summarized in Table S.1. As shown, a multivariate input window of 70 TRs (70×636 features, comprising cortical BOLD signals and head-motion parameters) is first processed by a stack of three 1D convolutional layers with increasing numbers of filters (64, 128, and 256), which extract short-range temporal features while preserving the temporal ordering of the input. The use of 70 time points reflects the fixed 50-s temporal window employed in this study, given the native HCP-YA repetition time (TR = 0.72 s). The resulting feature sequence is then passed directly to a GRU layer with 64 units, where recurrent hidden-state transitions and gating mechanisms (including update and reset gates) integrate information across the full temporal window. This recurrent processing enables the model to preserve temporally relevant features and capture delayed and cumulative respiratory effects distributed across the sequence. The GRU output is a fixed-length 64-dimensional representation, which is subsequently refined by a fully connected layer and mapped to a single scalar RV estimate via a linear output layer. Although cohort-specific hyperparameters differ across age groups, the architectural flow illustrated here is shared across all models and highlights the separation between local temporal feature extraction (CNN) and longer-range temporal dependency modeling through recurrent GRU-based integration.

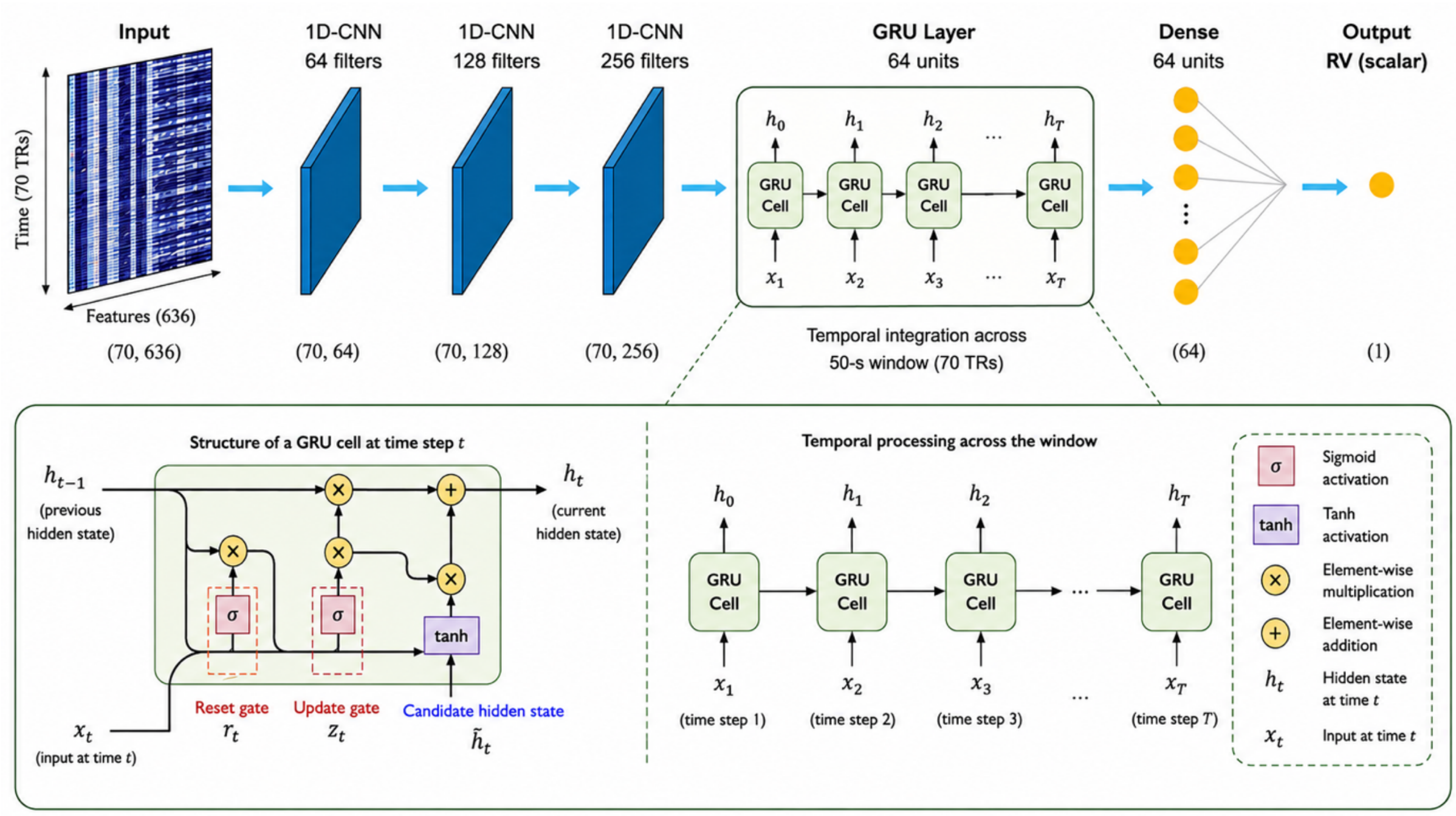

Figure S.1. Schematic illustration of the proposed 1D-CNN + GRU architecture for RV reconstruction in the HCP-YA cohort. A multivariate input window of 70 time points (70 ×636 features) is processed by three stacked 1D convolutional layers with increasing numbers of filters (64, 128, and 256), extracting short-range temporal features while preserving temporal ordering. The resulting feature sequence is then passed to a GRU layer with 64 units, where recurrent hidden-state transitions and gating mechanisms integrate information across the full temporal window to capture delayed and cumulative physiological effects. The GRU output is transformed into a fixed-length representation, which is subsequently refined by a fully connected layer and mapped to a single scalar RV estimate via a linear output layer. Numbers in parentheses denote the dimensionality of the intermediate representations at each stage.

2.2. Additional Design Considerations

2.2.1. Activation Function

Leaky ReLU was used instead of standard ReLU across all cohorts to address the "dying ReLU" issue, which can be especially problematic in deep or noise-sensitive architectures. By allowing a small gradient to flow for negative inputs, Leaky ReLU ensures continuous learning across all neurons and improves convergence reliability.

2.2.2. Batch Size Tuning

Batch size was adapted to the variability and data availability within each cohort. A smaller batch size (16) was selected for HCP-D and HCP-A to better handle greater physiological and motion heterogeneity, while a larger batch (32) improved training speed and stability in the more homogeneous HCP-YA group.

2.2.3. Regularization and Stability

Dropout was applied after each batch-normalized CNN layer to prevent overfitting. The higher dropout rate in children (0.4) accounts for increased physiological and signal

variability, while 0.3 was adequate for young and older adults. Batch normalization improved learning dynamics and allowed for more stable gradient propagation.

2.2.4. Temporal Dynamics with GRUs

GRUs enabled the network to retain critical temporal dependencies in the physiological signals. Compared to LSTMs, GRUs provide a more computationally efficient structure while offering similar performance in time-series modeling. GRU unit size was scaled according to temporal complexity, larger for children and smaller for aging adults.

3. Quality Assurance and Data Inclusion Criteria

We performed quality control on both the fMRI data and the accompanying physiological recordings to ensure reliable model training and evaluation.

For physiological signals, we systematically assessed respiratory and PPG traces for artifacts resulting from sensor malfunction, participant movement, suboptimal placement, or other acquisition-related issues. A standardized classification scheme (outlined in Section 1.2) was applied consistently across the HCP-D, HCP-YA, and HCP-A cohorts to identify and exclude low-quality signals. Only traces categorized as Class 1 (i.e., those with correctable spikes) were retained for further analysis.

For fMRI data, head motion was evaluated using framewise displacement. Scans with a mean displacement exceeding 1 mm were excluded to minimize motion-related confounds in the BOLD signal. Further details on physiological and motion quality assessment are provided in the following subsections.

3.1 Respiratory Signal Assessment

Respiratory trace quality was assessed for all scans in the three HCP cohorts using a seven-class artifact taxonomy. Figure S.1 illustrates example signals from each class, derived from the HCP-YA cohort. Although these examples are from a single cohort, similar artifact types were observed across HCP-D and HCP-A. Notably, Class 1 signals—those with correctable spikes—accounted for 22.7%, 26.8%, and 33.1% of scans in HCP-D, HCP-YA, and HCP-A, respectively (Table S.2). In contrast, severe artifacts such as clipping (Class 4), prolonged dropouts (Class 5), and complete signal loss (Class 7) were relatively rare but still present, highlighting the variability in data quality across the lifespan.

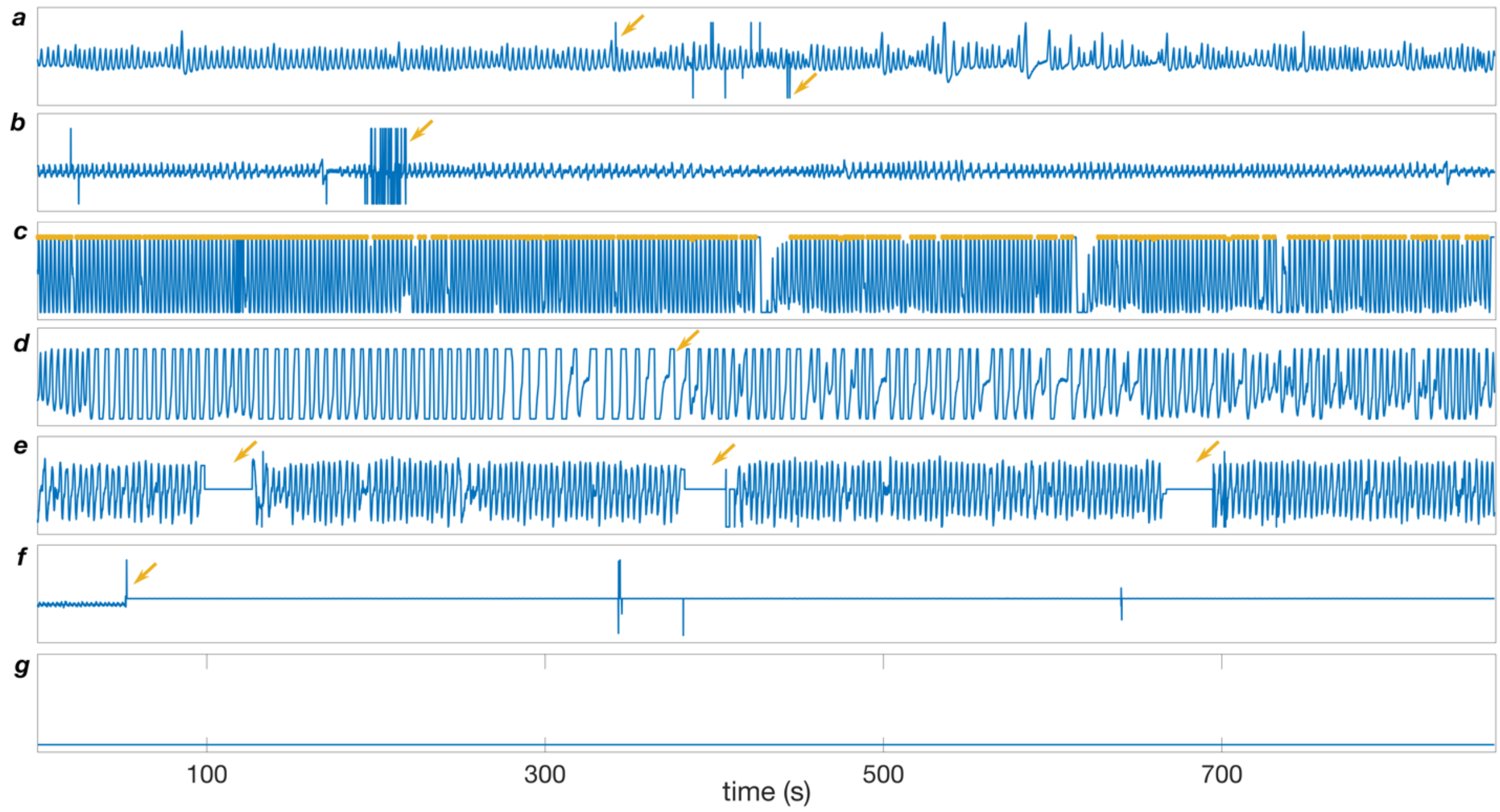

Figure S.2. Examples of respiratory signal artifacts in the HCP-YA dataset. (a) Respiratory signals with correctable spikes; (b) Respiratory signals with non-correctable spikes, indicating significant noise or artifacts; (c) Inhalations and exhalations peaking at maximum values, demonstrating extreme amplitude fluctuations; (d) Pronounced signal clipping, showing where the signal exceeds the recording capacity; (e) Periods without recording, marked by flatline sections due to signal loss or belt displacement; (f) Shifts in belt position leading to halted recording, evident by sudden signal drops; (g) Instances of non-recording, where no signal was captured. These examples highlight the variety and severity of artifacts that can compromise respiratory data quality in fMRI studies.

Table S.2. Classification of respiratory traces in the HCP dataset based on signal quality and artifact type (percent of scans).

	Number of scans	Class 1	Class 2	Class 3	Class 4	Class 5	Class 6	Class 7
HCP-D	2,451	22.7	27.3	35.6	7.8	5.3	0.7	0.6
HCP-YA	3,946	26.8	29.5	36.4	5.7	0.5	0.2	0.9
HCP-A	2,187	33.1	24.6	31.8	7.5	2.4	0.4	0.2

3.2 PPG Signal Assessment

PPG signals exhibited similar challenges, with artifacts ranging from correctable spikes to complete recording failure. A six-class categorization scheme was applied to all available PPG traces. Figure S.2 displays representative examples from the HCP-D dataset. Class 1 traces, which met quality criteria for model training, constituted 41.1%, 96.8%, and 37.0% of scans in the HCP-D, HCP-YA, and HCP-A cohorts, respectively (Table S.3). The high prevalence of usable signals in HCP-YA reflects better compliance and acquisition conditions, whereas lower rates in HCP-D and HCP-A may stem from age-specific challenges such as movement in children or poor peripheral perfusion in older adults.

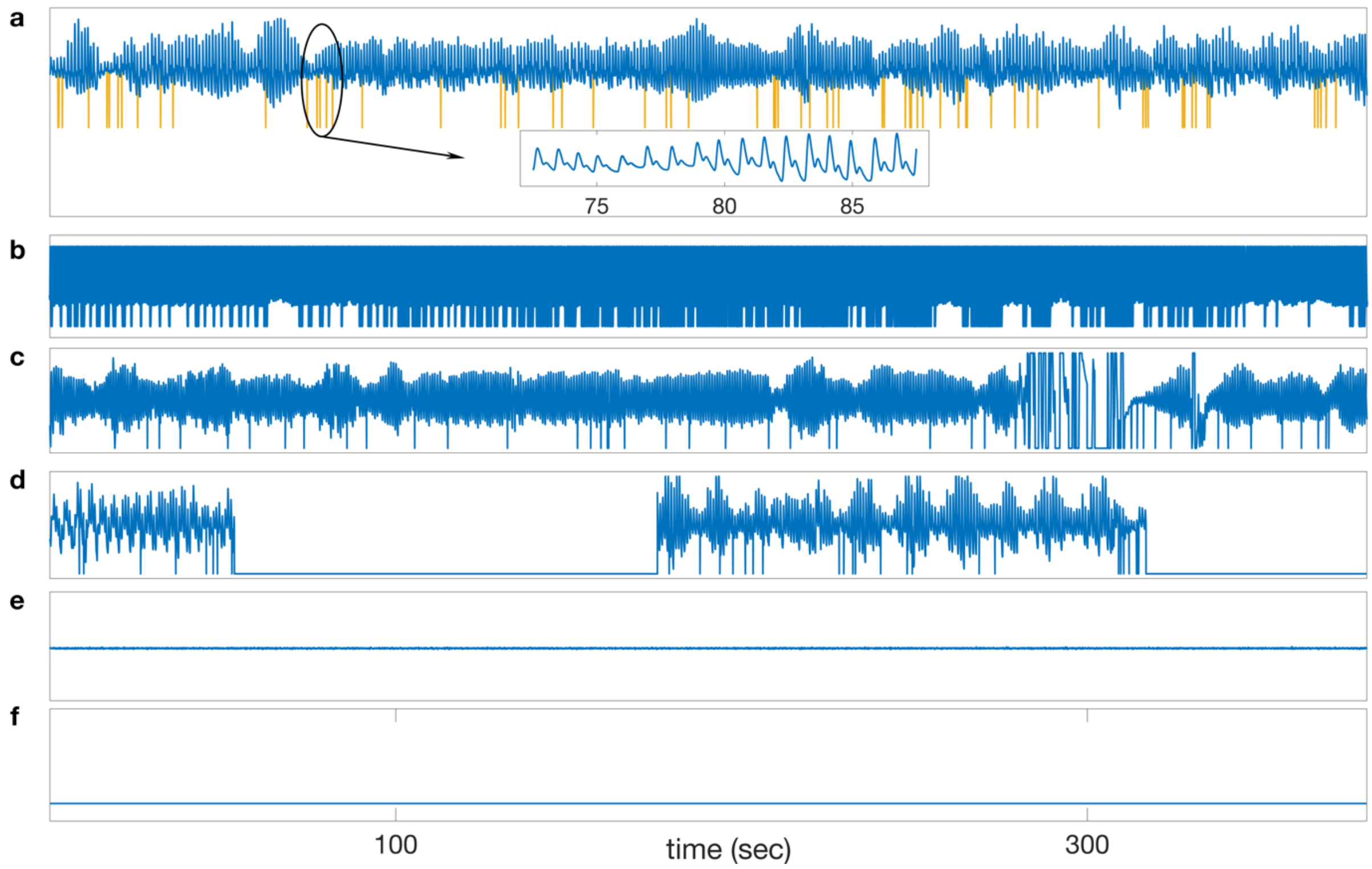

Figure S.3. Examples of cardiac signal artifacts in the HCP-D dataset. This figure displays typical PPG signal issues found in the HCP-D dataset, common in pediatric fMRI studies due to subject movement or technical challenges. Panel (a) shows a signal with correctable spikes. Panels (b)-(f) show discarded signals: (b) uncorrectable spikes, (c) signal clipping, (d) partial recording with gaps likely due to sensor disconnection, (e) very small amplitude, unlike a normal PPG signal, and (f) no recording. These examples highlight the variety and severity of artifacts that can compromise PPG data quality in fMRI studies.

Table S.3. Classification of PPG traces in the HCP dataset based on signal quality and artifact type (percent of scans).

	Number of scans	Class 1	Class 2	Class 3	Class 4	Class 5	Class 6
HCP-D	2,451	41.1	44.2	11.5	0.9	1.6	0.7
HCP-YA	3,946	96.8	1.8	0.9	0.1	0.3	0.1
HCP-A	2,187	37.0	52.7	8.2	0.4	1.2	0.5

3.3 Inclusion Criteria for Model Training

Only physiological signals classified as Class 1 (i.e., those with correctable spikes) were retained for model training. This conservative strategy ensured that the reconstructed RV and HRV waveforms were derived from high-quality, reliable ground-truth data. Importantly, inclusion required that both respiratory and PPG traces met the Class 1 criterion.

In addition to physiological signal quality, head motion was also carefully evaluated. Any scan with a mean framewise displacement exceeding 1 mm was excluded to minimize motion-related confounds in the BOLD signal. This dual-layer quality control—assessing

both physiological signal integrity and head motion—ensured that the final dataset was optimized for training robust machine learning models.

After applying all quality control procedures, including artifact removal, motion-based scan exclusion, and physiological trace validation (see Supplementary Materials, Section 3), the final datasets included:

- 550 resting-state fMRI scans from 411 participants (out of 2,451 total) in the HCP-D cohort,
- 1,050 scans from 590 individuals (out of 3,946 total) in the HCP-YA cohort, and
- 700 scans from 429 participants (out of 2,187 total) in the HCP-A cohort.

These curated datasets provided sufficient physiological fidelity and motion quality to support reliable training and evaluation of the proposed reconstruction models.

4. Cross-Validation Strategy

To evaluate model performance in a reliable and unbiased manner, we used 10-fold cross-validation, a widely accepted method in machine learning. This process is illustrated in Figures S.4 and S.5.

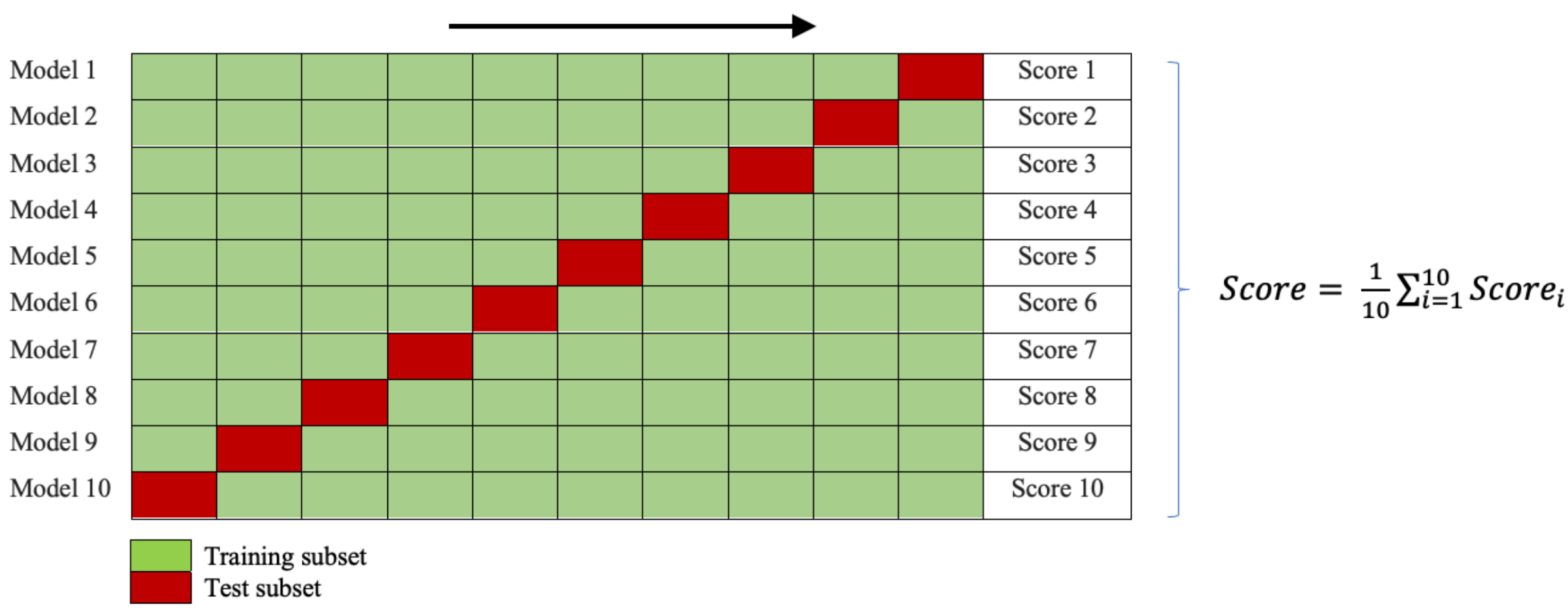

Figure S.4. Illustration of 10-fold cross-validation. The dataset is divided into 10 equal-sized subsets. In each iteration (fold), one subset is used for testing (red), while the remaining nine subsets are used for training (green). This process is repeated 10 times so that each subset serves as the test set exactly once. The final score is computed as the average performance across all 10 folds.

The full dataset was randomly divided into 10 equal-sized subsets (folds). To prevent data leakage, partitioning was performed at the subject level rather than the scan level, such that all scans from a given participant were assigned exclusively to a single fold. In each of the 10 iterations:

- 9 folds were used to train the model,
- 1 fold was held out for testing, and
- the testing fold rotated each time so that every sample was tested exactly once.

This process resulted in 10 trained models, each evaluated on a different test subset. Final performance metrics were calculated by averaging the results across all 10 folds, as shown in Figure S.3.

Within each training set (90% of the data), we further split off 20% for validation to monitor the model's performance during training and prevent overfitting. This inner split is illustrated in Figure S.5. In addition, early stopping based on validation loss was applied within each fold to halt training when performance on the validation set no longer improved, further reducing the risk of overfitting.

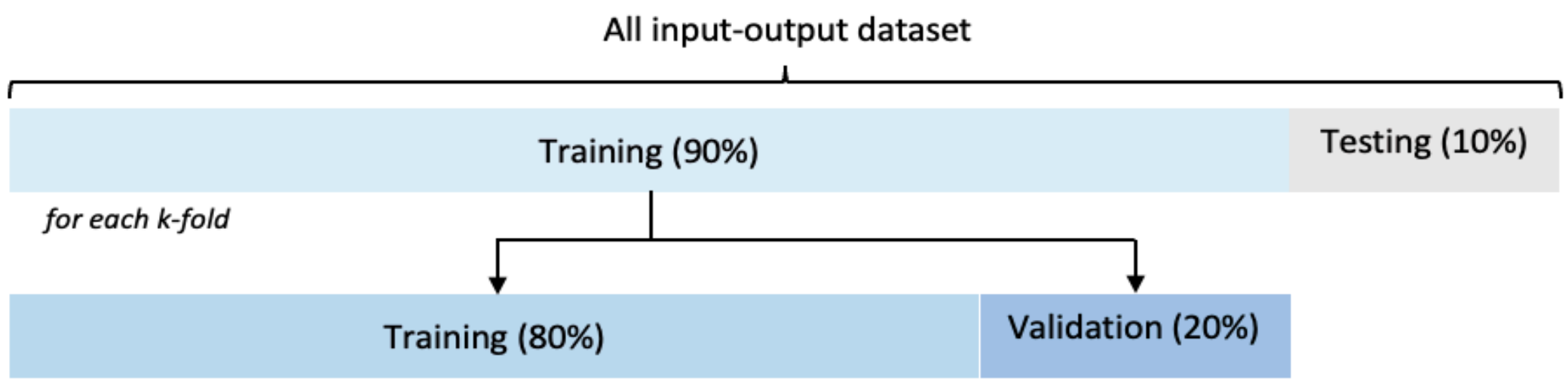

Figure S.5. Nested data split within each fold of the 10-fold cross-validation. For each training fold (90% of the data), 80% is used to train the model and 20% is held out as a validation set to monitor training progress and prevent overfitting. The outer 10% of the data is used only for testing.

This approach ensures that the model is evaluated on all available data, and that the reported performance metrics are stable and generalizable.

5. Evaluation of Model Performance

To evaluate the model's performance post-training, a multi-metric assessment strategy was implemented on the unseen test data derived from the ten-fold cross-validation. The model's predictive accuracy was quantified using the MAE, which measured the average magnitude of the errors in the predictions. In conjunction, the MSE was used to assess the impact of larger errors on the model's performance, which was particularly relevant when dealing with

significant physiological events in fMRI data. The Pearson correlation coefficient was applied to determine the linear correspondence between the predicted and actual respiratory and heart rate variation signals, indicating the model's capacity to track the overall trend of the signal. Furthermore, the DTW distance metric is employed to evaluate the temporal alignment of the predicted time-series with the target, a critical aspect for time-sequenced data like respiratory cycles and cardiac cycles. A lower DTW distance indicated a higher precision in the model's temporal predictions. Employing different metrics provided a robust validation of the model's capability to accurately reconstruct RV and HRV, which was essential for the reliable analysis of fMRI data. In these investigations, the Friedman test was employed to rigorously assess the significance of discrepancies in the accuracy of RV and HRV reconstructions across various inputs and machine learning models, confirming statistically significant differentiations in performance.

6. Technical Specifications and Computational Environment

The deep learning model employed in this study was configured with a learning rate (η) of 0.001, utilizing the Adam optimizer for efficient training convergence (Kingma & Ba, 2014). The deep learning architecture employed in this study was designed and assessed using Python, leveraging the Keras framework with TensorFlow serving as the underlying backend. The computational analyses pertinent to this segment were executed on the Advanced Research Computing (ARC) cluster housed at the University of Calgary. This environment provided two processors, supplemented with 200 GB of RAM, facilitating efficient network training. The code of the proposed method is available on GitHub (https://github.com/jaliladde).

7. Representative RV and HRV Waveform Reconstruction Examples

Figure S.6 presents representative RV reconstruction examples from four test scans in the HCP-YA cohort, illustrating model performance across varying physiological conditions. Panels a and b demonstrate high-quality reconstructions, where the model accurately captured the temporal dynamics and amplitude fluctuations of the measured RV signal. Reconstruction performance was particularly strong during periods of pronounced respiratory variability, likely because respiration-induced fluctuations produced clearer modulation patterns in both the BOLD signal and head motion parameters. The inclusion of motion parameters further

improved reconstruction accuracy, consistent with their ability to capture respiration-related pseudomotion and subtle breathing-associated head movements.

To provide a balanced evaluation, panels c and d illustrate representative lower-performance cases. In these scans, the measured RV signals exhibited substantially lower amplitude and reduced temporal variability, resulting in weaker physiological modulation of the BOLD signal. Under such conditions, respiration-related fluctuations become more difficult to distinguish from background noise and other non-neuronal signal sources, reducing reconstruction accuracy. In addition, flatter respiratory patterns provide less informative temporal structure for the model to learn and reconstruct. Despite these challenges, the reconstructed RV waveforms continued to capture portions of the underlying temporal trends, demonstrating the robustness of the proposed framework across a range of physiological signal characteristics.

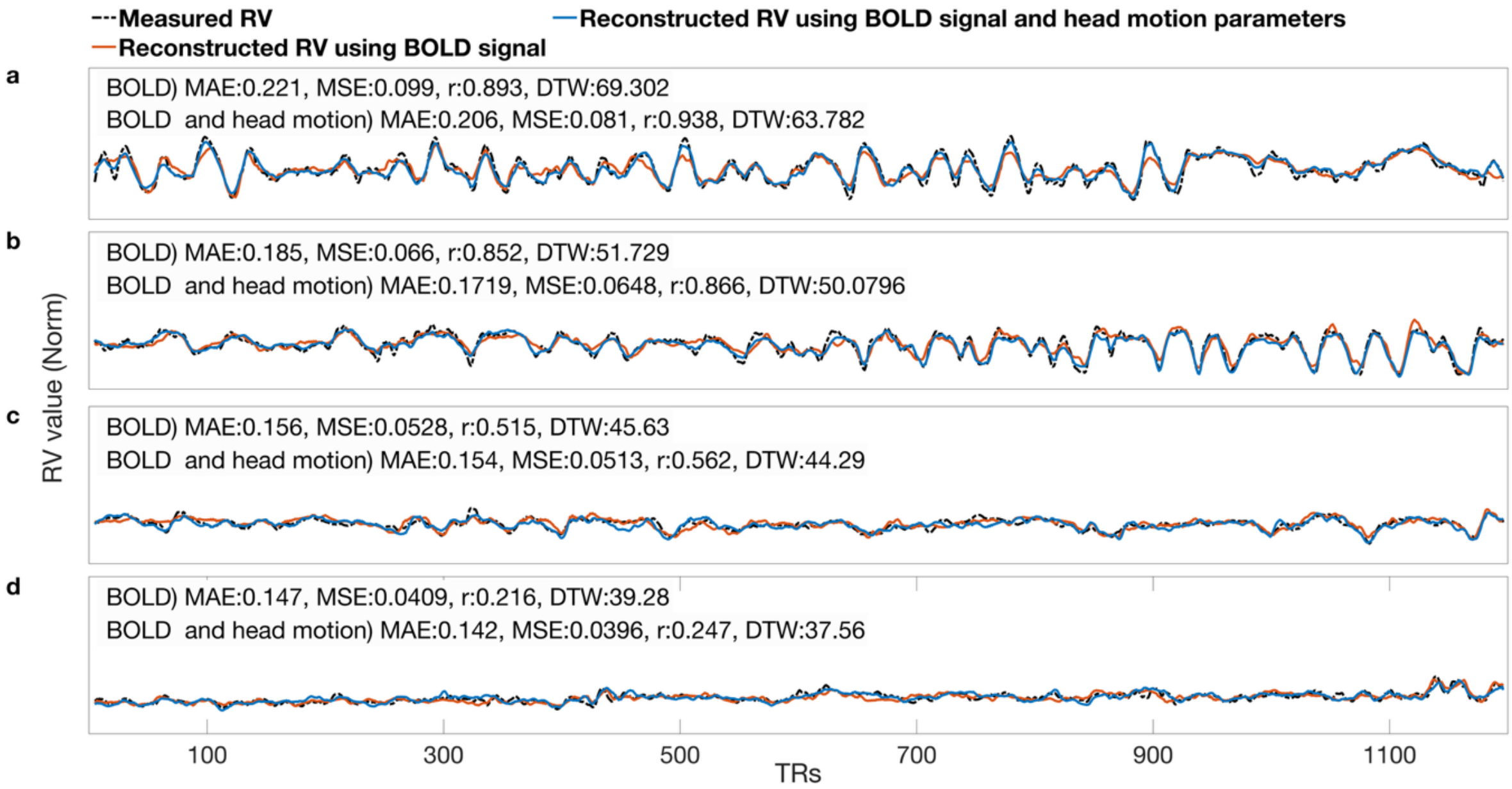

Figure S.6. Representative examples of RV waveform reconstruction using different input configurations. Measured RV signals (black) are compared with reconstructed RV waveforms using mean BOLD signals from 630 ROIs (blue) and a combination of BOLD signals and head motion parameters (red) across four test scans (panels a–d). For each case, performance metrics—MAE, MSE, Pearson correlation coefficient (r), and DTW—are reported. The addition of head motion parameters consistently improves reconstruction accuracy, particularly during periods of large respiratory fluctuations, demonstrating the model's capacity to integrate motion-related information for enhanced physiological signal estimation.

Figure S.7 shows representative HRV waveform reconstructions from four HCP-YA test subjects. The results show that the model effectively reconstructed HRV dynamics, especially during periods of marked physiological fluctuation.

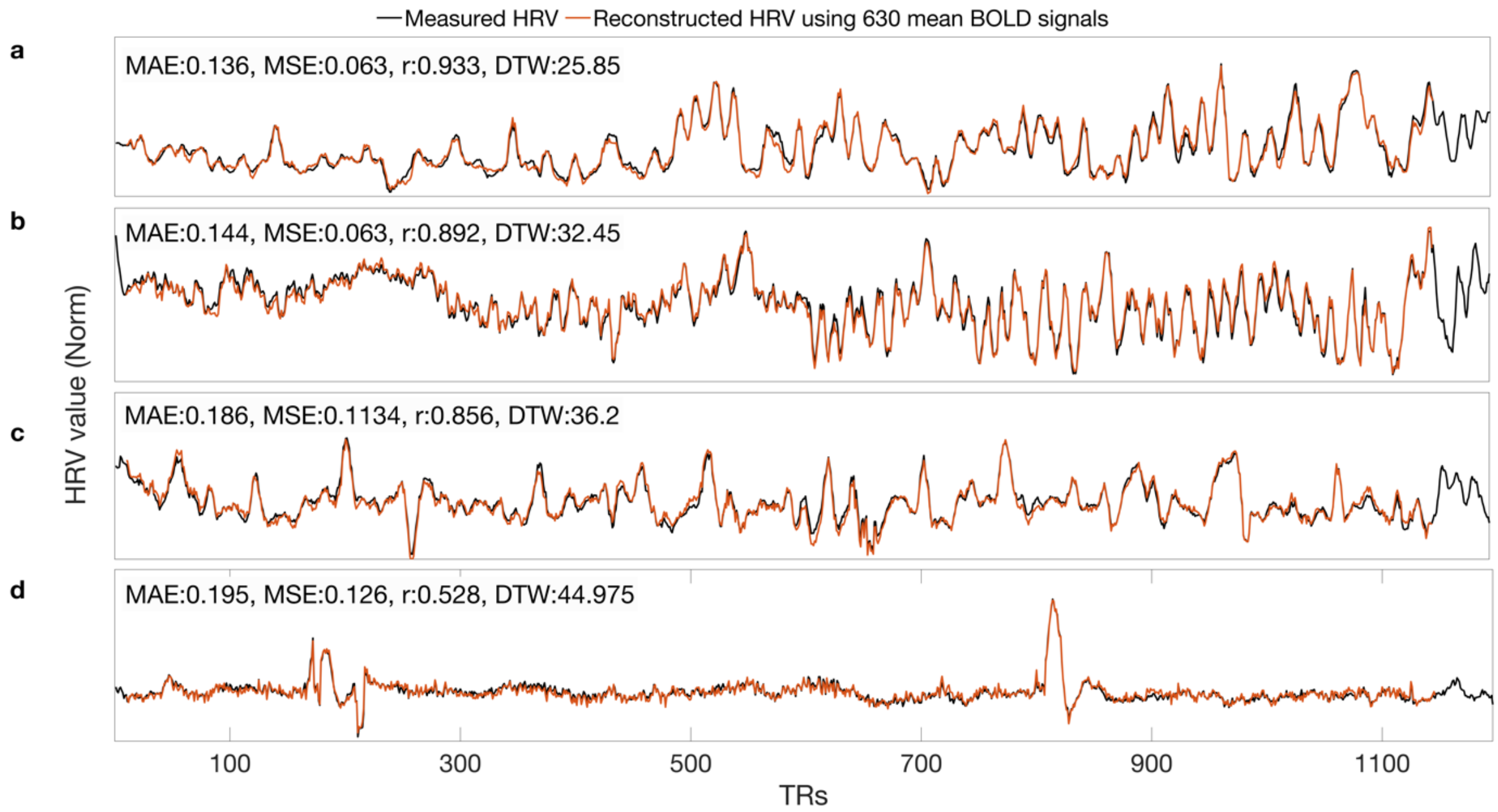

Figure S.7. Representative examples of HRV waveform reconstruction using mean BOLD signals. Measured HRV signals (black) are compared with reconstructed HRV waveforms (orange) across four test scans (panels a–d). Reconstructions were performed using mean BOLD signals from 630 ROIs without head motion parameters, as their inclusion did not improve HRV estimation. For each subject, performance metrics including MAE, MSE, Pearson correlation coefficient (r), and DTW are provided. The model accurately captured temporal HRV fluctuations, with higher fidelity observed during periods of increased physiological variability.

8. Spectral Content of Measured RV and HRV at Native Temporal Resolution

To characterize the frequency content of physiological signals used in this study and to motivate the use of native temporal resolution without band-limited filtering, we analyzed the power spectral density (PSD) of the RV and HRV signals across the three HCP cohorts: HCP-D, HCP-YA, and HCP-A.

Supplementary Figures S.8 and S.9 show the group-level PSDs for RV and HRV, respectively, computed from the measured physiological recordings using the native sampling rates of each dataset. For visualization clarity, PSDs are presented on both linear and logarithmic power scales. Shaded regions indicate inter-subject variability, and dashed vertical lines mark the commonly used upper cutoff frequency of 0.15 Hz employed in prior reconstruction studies based on temporally downsampled fMRI data.

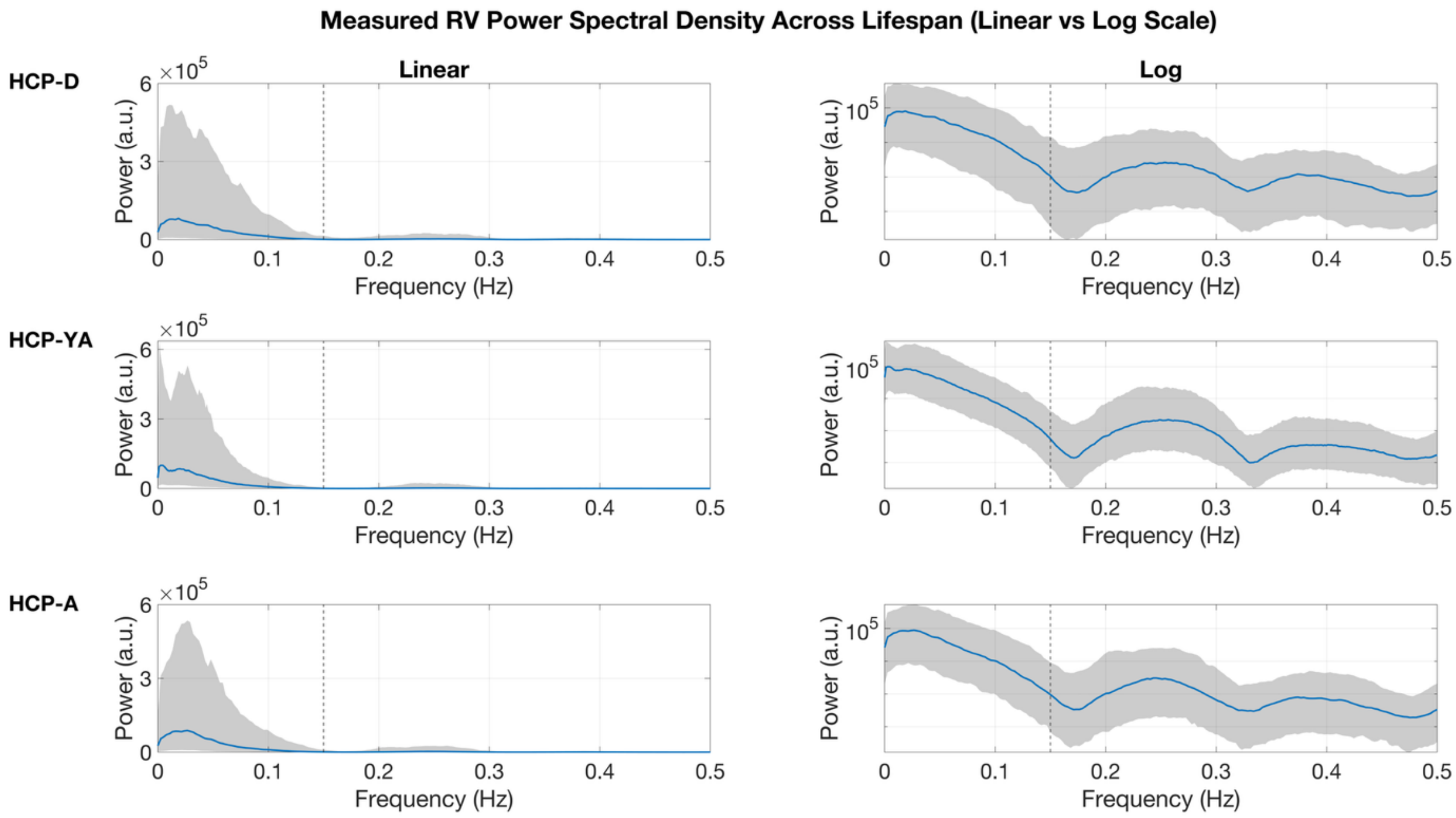

Figure S.8. Measured RV Power Spectral Density Across the Lifespan. Group-level PSD of measured RV for the HCP-D, HCP-YA, and HCP-A cohorts. PSDs are shown on both linear (left) and logarithmic (right) power scales to highlight dominant low-frequency components and lower-amplitude higher-frequency content. Solid lines denote the cohort-averaged PSD, and shaded regions indicate inter-subject variability. The vertical dashed line marks 0.15 Hz, corresponding to the upper frequency cutoff commonly used in prior fMRI-based RV reconstruction studies. Across all cohorts, non-negligible RV spectral power extends beyond 0.15 Hz when analyzed at native temporal resolution.

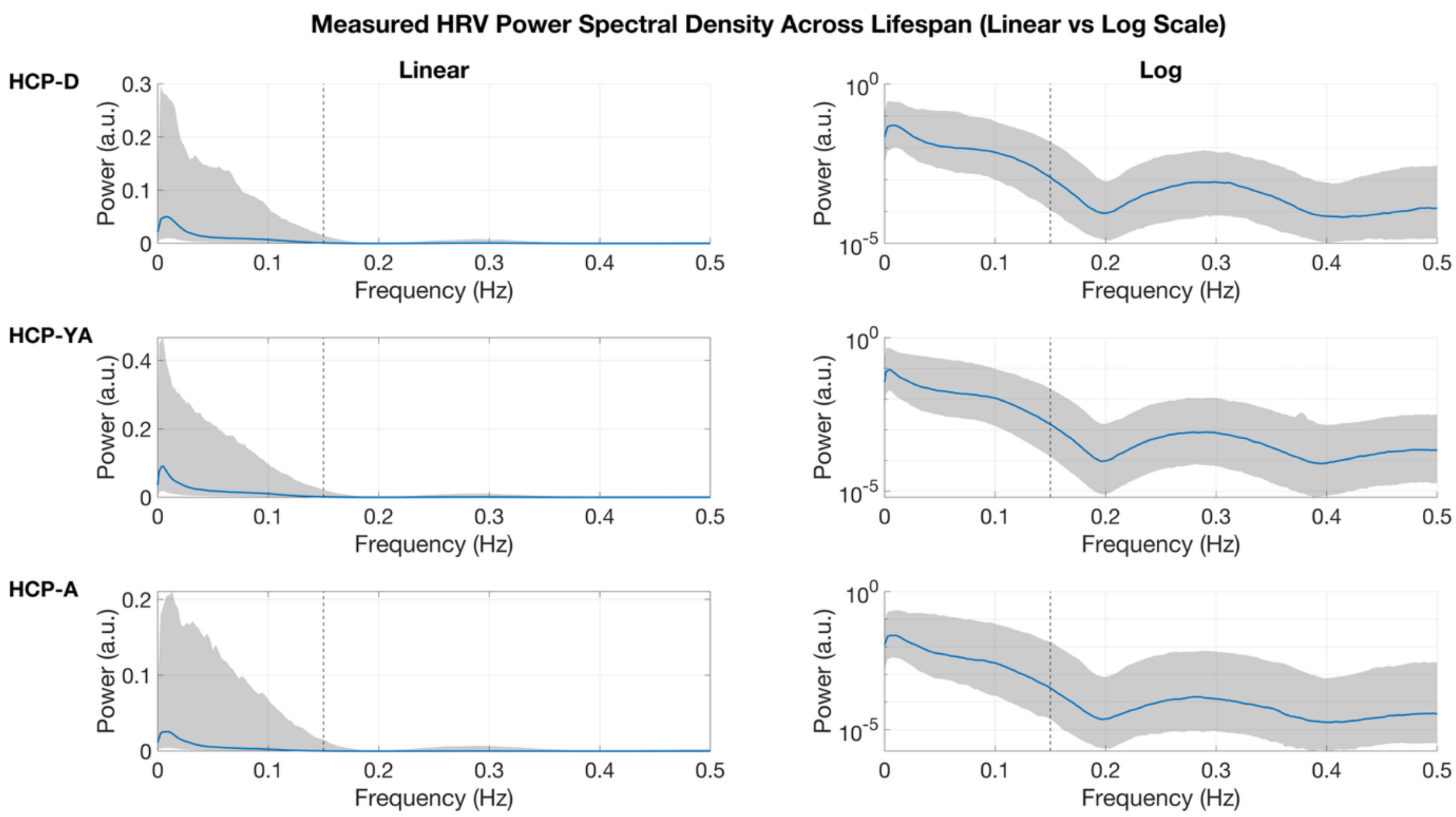

Figure S.9. Measured HRV Power Spectral Density Across the Lifespan. Group-level PSD of measured HRV for the HCP-D, HCP-YA, and HCP-A cohorts, displayed using linear (left) and logarithmic (right) power scales. Solid lines represent cohort-averaged PSDs, with shaded regions indicating inter-subject variability. The vertical dashed line denotes 0.15 Hz, a commonly applied upper cutoff in previous HRV reconstruction approaches based on temporally downsampled fMRI data. While low-frequency components dominate, measurable HRV spectral power persists above 0.15 Hz across all cohorts, particularly evident in the log-scale representation.

Across all cohorts, RV spectra exhibit dominant low-frequency components below approximately 0.1 Hz, consistent with slow breathing fluctuations. However, clear and non-negligible spectral power is also observed above 0.15 Hz, particularly in the log-scale representations, indicating the presence of faster respiratory modulations and transient dynamics that are not captured when signals are restricted to narrow low-frequency bands. This pattern is consistently observed in HCP-D, HCP-YA, and HCP-A, despite differences in age and physiological variability.

A similar trend is evident for HRV. While the majority of HRV power is concentrated at low frequencies, measurable spectral content persists beyond 0.15 Hz across all cohorts. These higher-frequency components likely reflect beat-to-beat variability and rapid autonomic fluctuations that are physiologically meaningful but are attenuated or removed by temporal downsampling and low-pass filtering strategies commonly adopted in earlier fMRI-based reconstruction approaches.

Importantly, these spectral characteristics are largely obscured in linear-scale PSD plots due to the dominance of low-frequency power, but become clearly visible in logarithmic representations. This observation underscores the value of log-scale visualization for assessing physiologically relevant high-frequency content in RV and HRV signals.

Together, these results demonstrate that both RV and HRV contain substantial spectral information beyond the conventional 0.01-0.15 Hz band when analyzed at native temporal resolution. Consequently, reconstruction methods that operate directly on native-TR fMRI data without frequency-restricted filtering are better positioned to preserve the full temporal bandwidth of physiological fluctuations. This provides a principled motivation for the design choices adopted in the present study and supports the interpretation that downsampling and aggressive low-pass filtering may discard physiologically meaningful information relevant for accurate RV and HRV reconstruction.

9. Impact of Machine Learning Architecture in Developmental and Aging Cohorts

Tables S.4 and S.5 summarize the performance of the proposed 1D-CNN + GRU framework relative to previously published CNN- and RNN-based architectures on the HCP-D and HCP-A cohorts, respectively. Across both developmental and aging populations, the proposed model consistently achieved the lowest reconstruction error (MAE and MSE), the highest temporal agreement (Pearson's r), and improved temporal alignment (DTW) for both RV and

HRV under matched preprocessing, temporal resolution, and input conditions. While CNN-only and bi-LSTM baselines achieved competitive performance, they exhibited systematically reduced accuracy across all metrics relative to the proposed architecture. These performance differences were statistically significant across architectures (Friedman test, $p < 0.001$) and mirrored the trends observed in the young adult cohort, demonstrating that the performance gains of the 1D-CNN + GRU framework generalize across age groups and are robust to age-related variability in physiological dynamics and BOLD signal properties.

Table S.4. Comparison of RV and HRV reconstruction performance across different machine learning architectures on the HCP-D dataset. All models were trained and evaluated using the native temporal resolution (TR = 0.8 s) without temporal downsampling and with matched input features. Window-based models were evaluated using a fixed temporal context of 50 s, whereas the bi-LSTM model operates in a sequence-to-sequence manner over the full fMRI time series.

Method	Temporal Context	Signal	MAE	MSE	r	DTW
1D-CNN + GRU (Proposed)	50 s	RV	0.247 ± 0.0053	0.118 ± 0.0081	0.629 ± 0.039	24.57 ± 0.90
		HRV	0.232 ± 0.0060	0.105 ± 0.0072	0.612 ± 0.046	16.28 ± 0.61
1D-CNN Addeh et al. (2025)	50 s	RV	0.269 ± 0.0071	0.131 ± 0.0036	0.586 ± 0.057	26.12 ± 0.87
		HRV	0.251 ± 0.0045	0.115 ± 0.0050	0.571 ± 0.029	17.16 ± 0.64
1D-CNN Salas et al. (2021)	50 s	RV	0.272 ± 0.0071	0.133 ± 0.0042	0.582 ± 0.049	26.49 ± 0.62
		HRV	0.255 ± 0.0045	0.118 ± 0.0027	0.568 ± 0.036	17.31 ± 0.72
bi-LSTM Bayrak et al. (2025)	Full sequence	RV	0.268 ± 0.0049	0.126 ± 0.0065	0.591 ± 0.074	25.83 ± 0.46
		HRV	0.249 ± 0.0043	0.112 ± 0.0054	0.582 ± 0.032	17.21 ± 0.54

Table S.5. Comparison of RV and HRV reconstruction performance across different machine learning architectures on the HCP-A dataset. All models were trained and evaluated using the native temporal resolution (TR = 0.8 s) without temporal downsampling and with matched input features. Window-based models were evaluated using a fixed temporal context of 50 s, whereas the bi-LSTM model operates in a sequence-to-sequence manner over the full fMRI time series.

Method	Temporal Context	Signal	MAE	MSE	r	DTW
1D-CNN + GRU (Proposed)	50 s	RV	0.254 ± 0.0046	0.133 ± 0.0074	0.624 ± 0.026	23.92 ± 0.70
		HRV	0.231 ± 0.0084	0.115 ± 0.0067	0.616 ± 0.038	16.85 ± 0.92
1D-CNN Addeh et al. (2025)	50 s	RV	0.276 ± 0.0077	0.145 ± 0.0063	0.586± 0.041	25.79 ± 0.57
		HRV	0.254 ± 0.0053	0.126 ± 0.0091	0.571 ± 0.067	18.22 ± 0.82
1D-CNN Salas et al. (2021)	50 s	RV	0.283 ± 0.0065	0.149 ± 0.0072	0.582± 0.055	25.86 ± 0.63
		HRV	0.257 ± 0.0081	0.129 ± 0.0079	0.568 ± 0.061	18.31 ± 0.90
bi-LSTM Bayrak et al. (2025)	Full sequence	RV	0.272 ± 0.0051	0.143 ± 0.0052	0.591± 0.036	25.30 ± 0.49
		HRV	0.248 ± 0.0076	0.122 ± 0.0086	0.575 ± 0.040	17.94 ± 0.65

10. Quality Assessment of Physiological Data in New Datasets

Figures S.10 to S.13 provide further examples of corrupted physiological signals from the two new datasets, Dataset 1 and Dataset 2, reiterating the challenges discussed in the main paper regarding the acquisition of high-quality respiratory and PPG data in fMRI studies.

These examples reinforce the difficulty in obtaining reliable respiratory and PPG signals, even when data collection is attempted, which can significantly impact the effectiveness of physiological confound correction methods.

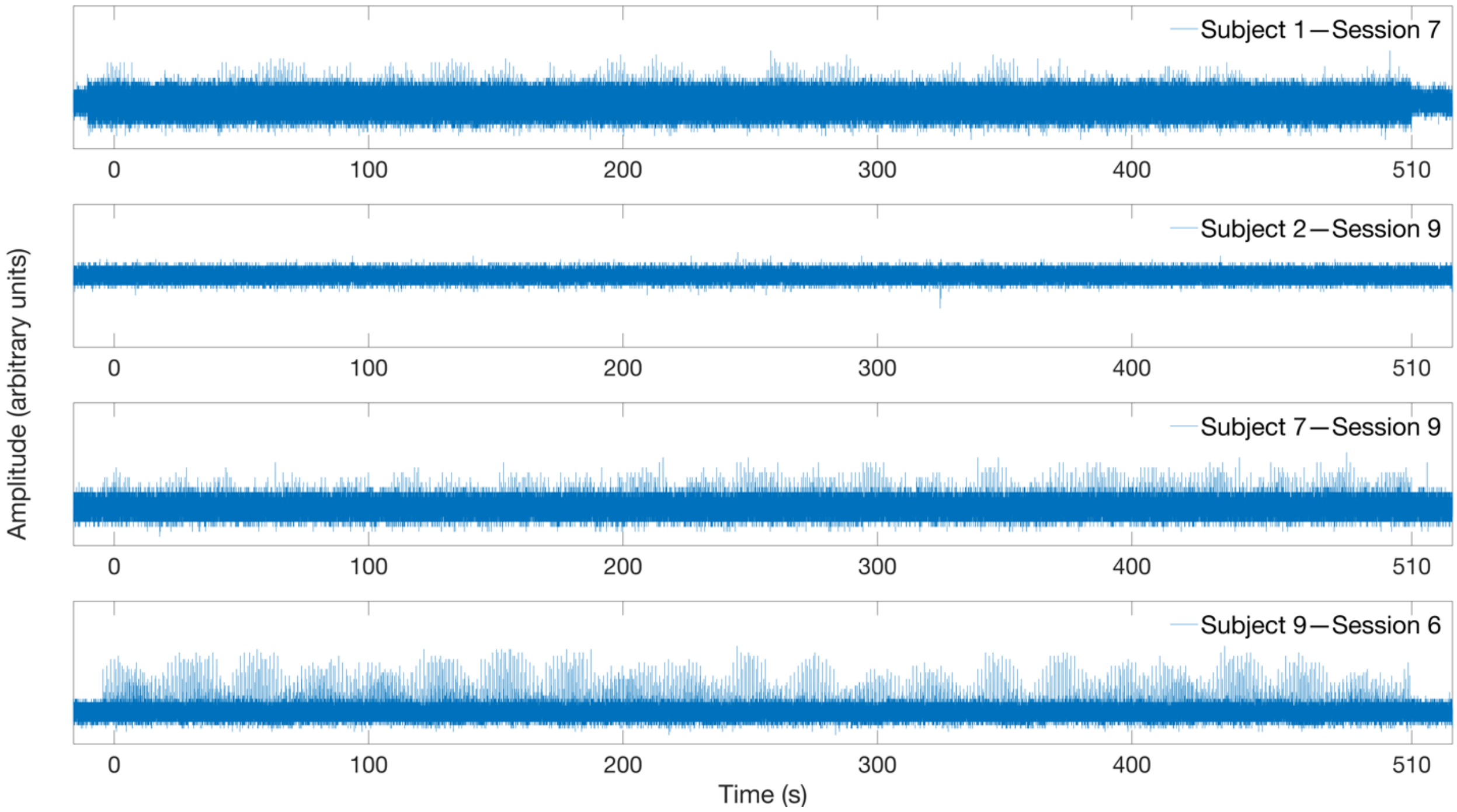

Figure S.10. Examples of corrupted respiratory data in Dataset 1 (Zvolanek et al., 2023)

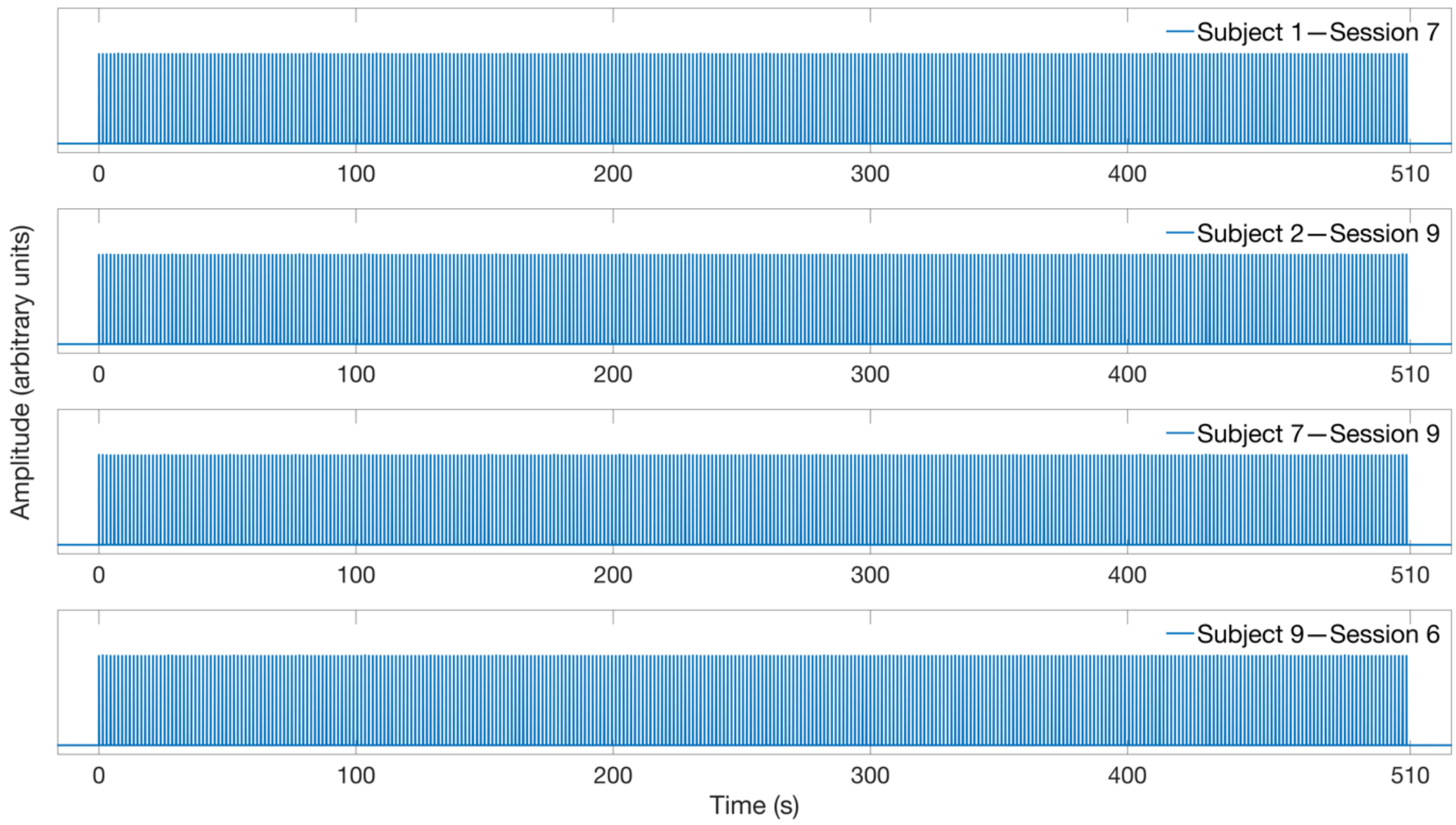

Figure S.11. Examples of corrupted PPG data in Dataset 1 (Zvolanek et al., 2023)

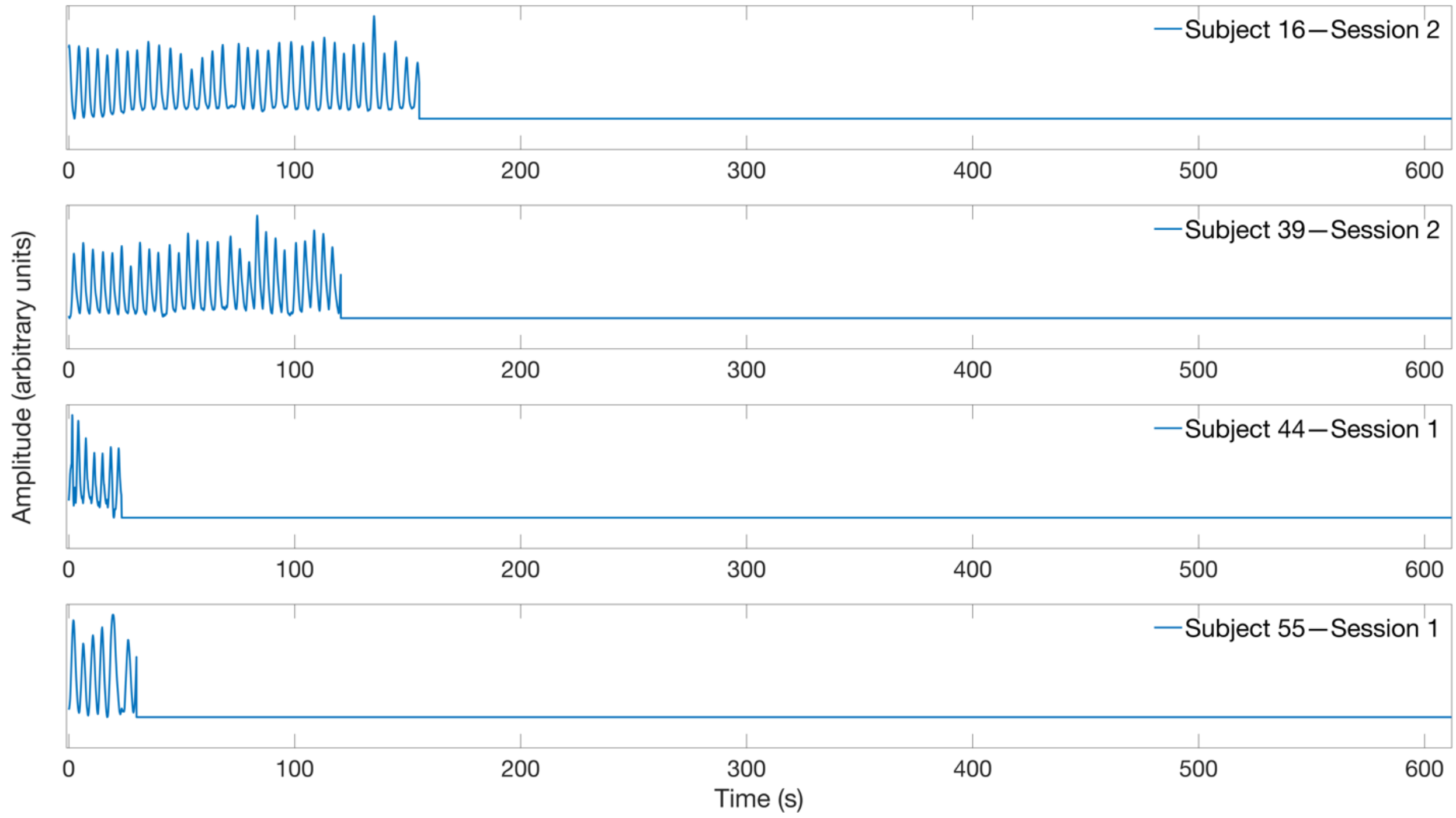

Figure S.12. Examples of corrupted respiratory data in Dataset 2. Although the scan duration was 612 seconds, the respiratory data was only partially recorded (Spreng et al., 2022)

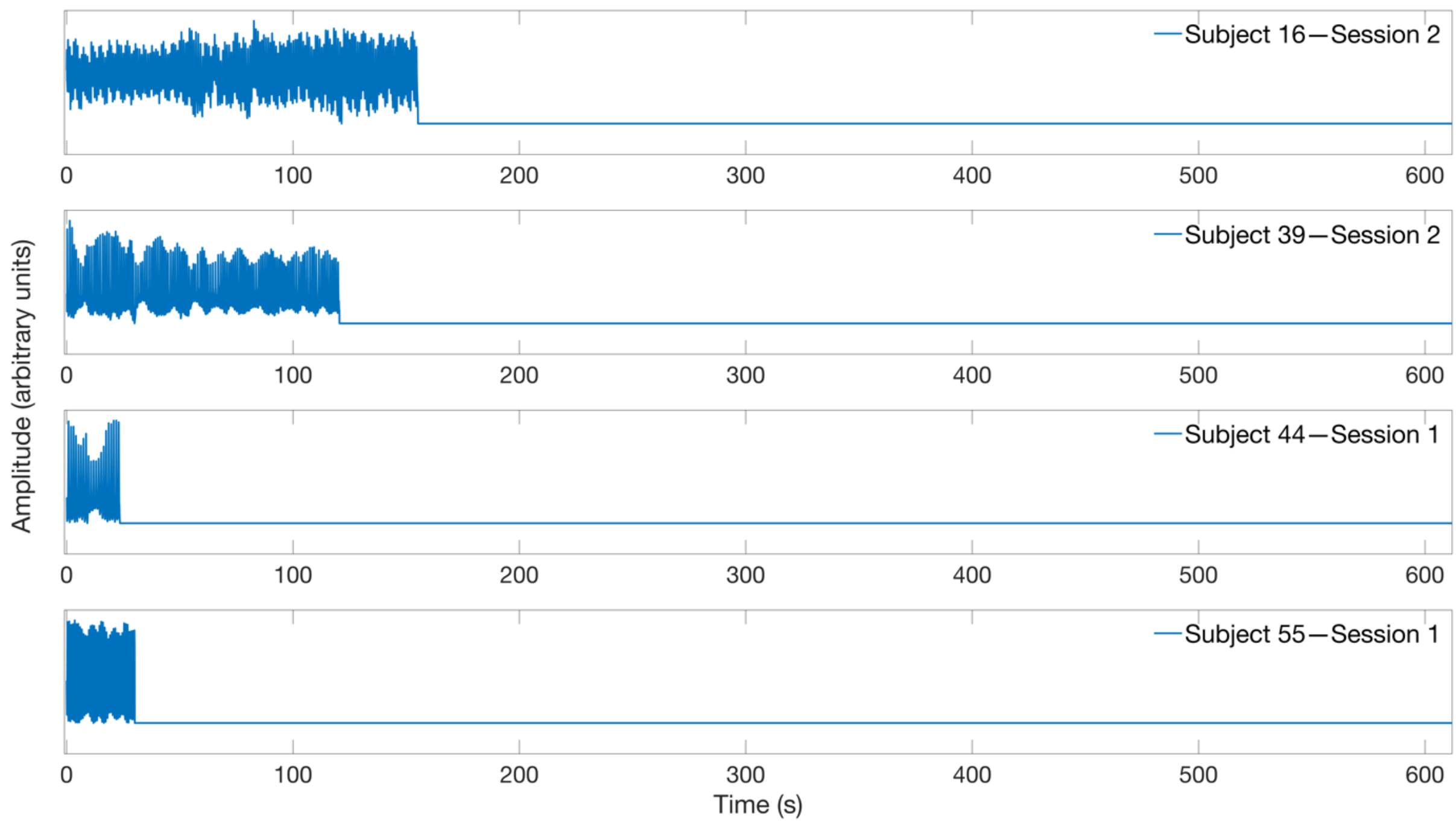

Figure S.13. Examples of corrupted PPG data in Dataset 2. Although the scan duration was 612 seconds, the respiratory data was only partially recorded (Spreng et al., 2022)

11. Independent Dataset Evaluation

Table S.6 summarizes quantitative RV and HRV reconstruction performance for the two independent datasets evaluated in this study. Higher correlation values are observed for Dataset 1, which includes a breath-hold task that induces large and regular respiratory fluctuations, whereas lower correlations are observed for Dataset 2, which consists of resting-state data with more irregular breathing patterns and lower temporal resolution. Error-based and temporal alignment metrics (MAE, MSE, and DTW) follow consistent trends across datasets.

Table S.6. RV and HRV reconstruction performance on independent datasets

Dataset	Signal	MAE	MSE	r	DTW
Dataset 1 (BH, TR=1.5s)<br>Without head motion	RV	0.245	0.116	0.81	20.26
Dataset 1 (BH, TR=1.5s)	HRV				
Dataset 1 (BH, TR=1.5s)<br>With head motion	RV	0.213	0.110	0.83	18.11
Dataset 2 (Rest, TR=3s)<br>Without head motion	RV	0.287	0.131	0.55	13.34
Dataset 2 (Rest, TR=3s)	HRV				
Dataset 2 (Rest, TR=3s)<br>With head motion	RV	0.274	0.128	0.57	12.56

12. Effect of Temporal Window Directionality on HRV Reconstruction

To evaluate the impact of temporal window directionality on HRV reconstruction, we compared model performance using past-only, future-only, and bidirectional (past + future) temporal windows. Performance was quantified using the Pearson correlation coefficient between reconstructed and measured HRV time series, averaged across subjects for each dataset.

As summarized in Table S.7, bidirectional windows consistently achieved the highest reconstruction accuracy across all cohorts. Importantly, models using future-only windows outperformed those using past-only windows in all three datasets. This asymmetry is consistent with the delayed hemodynamic manifestation of cardiac-related fluctuations in the BOLD signal, whereby BOLD responses at later time points encode information about earlier cardiac activity more directly than preceding samples.

These results support the use of bidirectional temporal context for HRV reconstruction and explain the reduced accuracy observed when restricting the model to unidirectional windows.

Table S.7. HRV reconstruction performance using unidirectional and bidirectional temporal windows

Dataset	Only-past	Both-side	Only-future
HCP-D	0.493 ± 0.066	0.613 ± 0.046	0.547 ± 0.081
HCP-YA	0.537 ± 0.074	0.642 ± 0.038	0.584 ± 0.069
HCP-A	0.527 ± 0.038	0.617 ± 0.077	0.572 ± 0.053

References

Addeh, A., Vega, F., Morshedi, A., Williams, R. J., Pike, G. B., & MacDonald, M. E. (2025). Machine learning-based estimation of respiratory fluctuations in a healthy adult population using resting state BOLD fMRI and head motion parameters. *Magnetic Resonance in Medicine*, *93*(3), 1365-1379.

Andersson, J. L., Skare, S., & Ashburner, J. (2003). How to correct susceptibility distortions in spin-echo echo-planar images: application to diffusion tensor imaging. *NeuroImage*, *20*(2), 870-888. https://doi.org/10.1016/s1053-8119(03)00336-7

Avants, B. B., Tustison, N. J., Song, G., Cook, P. A., Klein, A., & Gee, J. C. (2011). A reproducible evaluation of ANTs similarity metric performance in brain image registration. *NeuroImage*, *54*(3), 2033-2044. https://doi.org/https://doi.org/10.1016/j.neuroimage.2010.09.025

Bayrak, R. G., Hansen, C. B., Salas, J. A., Ahmed, N., Lyu, I., Mather, M., Huo, Y., & Chang, C. (2025). DeepPhysioRecon: Tracing peripheral physiology in low frequency fMRI dynamics. *Imaging Neuroscience*, *3*, IMAG. a. 163.

Chang, C., Cunningham, J. P., & Glover, G. H. (2009). Influence of heart rate on the BOLD signal: the cardiac response function. *NeuroImage*, *44*(3), 857-869.

Harms, M. P., Somerville, L. H., Ances, B. M., Andersson, J., Barch, D. M., Bastiani, M., Bookheimer, S. Y., Brown, T. B., Buckner, R. L., Burgess, G. C., Coalson, T. S., Chappell, M. A., Dapretto, M., Douaud, G., Fischl, B., Glasser, M. F., Greve, D. N., Hodge, C., Jamison, K. W., . . . Yacoub, E. (2018). Extending the Human Connectome Project across ages: Imaging protocols for the Lifespan Development and Aging projects. *NeuroImage*, *183*, 972-984. https://doi.org/10.1016/j.neuroimage.2018.09.060

Jenkinson, M., Bannister, P., Brady, M., & Smith, S. (2002). Improved Optimization for the Robust and Accurate Linear Registration and Motion Correction of Brain Images. *NeuroImage*, *17*(2), 825-841. https://doi.org/https://doi.org/10.1006/nimg.2002.1132

Kingma, D., & Ba, J. (2014). Adam: A Method for Stochastic Optimization. Proceedings of the 3rd International Conference on Learning Representations (ICLR), San Diego, CA, USA.

Salas, J. A., Bayrak, R. G., Huo, Y., & Chang, C. (2021). Reconstruction of respiratory variation signals from fMRI data. *NeuroImage*, *225*, 117459. https://doi.org/https://doi.org/10.1016/j.neuroimage.2020.117459

Smith, S. M., Beckmann, C. F., Andersson, J., Auerbach, E. J., Bijsterbosch, J., Douaud, G., Duff, E., Feinberg, D. A., Griffanti, L., Harms, M. P., Kelly, M., Laumann, T., Miller, K. L., Moeller, S., Petersen, S., Power, J., Salimi-Khorshidi, G., Snyder, A. Z., Vu, A. T., . . . Glasser, M. F. (2013). Resting-state fMRI in the Human Connectome Project. *NeuroImage*, *80*, 144-168. https://doi.org/10.1016/j.neuroimage.2013.05.039

Spreng, R. N., Setton, R., Alter, U., Cassidy, B. N., Darboh, B., DuPre, E., Kantarovich, K., Lockrow, A. W., Mwilambwe-Tshilobo, L., & Luh, W.-M. (2022). Neurocognitive aging data release with behavioral, structural and multi-echo functional MRI measures. *Scientific Data*, *9*(1), 119.

Van Essen, D. C., Smith, S. M., Barch, D. M., Behrens, T. E., Yacoub, E., Ugurbil, K., & Consortium, W.-M. H. (2013). The WU-Minn human connectome project: an overview. *NeuroImage*, *80*, 62-79.

Zvolanek, K. M., Moia, S., Dean, J. N., Stickland, R. C., Caballero-Gaudes, C., & Bright, M. G. (2023). Comparing end-tidal CO2, respiration volume per time (RVT), and average gray matter signal for mapping cerebrovascular reactivity amplitude and delay with breath-hold task BOLD fMRI. *NeuroImage*, *272*, 120038.
